# Upstream open reading frames regulate translation of cancer-associated transcripts and encode HLA-presented immunogenic tumor antigens

**DOI:** 10.1007/s00018-022-04145-0

**Published:** 2022-03-03

**Authors:** Annika Nelde, Lea Flötotto, Lara Jürgens, Laura Szymik, Elvira Hubert, Jens Bauer, Christoph Schliemann, Torsten Kessler, Georg Lenz, Hans-Georg Rammensee, Juliane S. Walz, Klaus Wethmar

**Affiliations:** 1 Clinical Collaboration Unit Translational Immunology, Department of Internal Medicine, German Cancer Consortium (DKTK), University Hospital Tübingen, Otfried-Müller-Str. 10, 72076 Tübingen, Germany; 2 Department of Immunology, Institute for Cell Biology, University of Tübingen, 72076 Tübingen, Germany; 3 Cluster of Excellence iFIT (EXC2180) “Image-Guided and Functionally Instructed Tumor Therapies”, University of Tübingen, 72076 Tübingen, Germany; 4 Department of Medicine A, Hematology, Oncology, Hemostaseology and Pneumology, University Hospital Münster, Albert-Schweitzer-Campus 1A, 48149 Münster, Germany; 5 German Cancer Consortium (DKTK) and German Cancer Research Center (DKFZ), Partner Site Tübingen, 72076 Tübingen, Germany; 6 Dr. Margarete Fischer-Bosch Institute of Clinical Pharmacology, Robert Bosch Center for Tumor Diseases (RBCT), 70376 Stuttgart, Germany

**Keywords:** Cancer, Immunopeptidomics, Mass spectrometry, Translational control, uORF

## Abstract

**Background:**

Upstream open reading frames (uORFs) represent translational control elements within eukaryotic transcript leader sequences. Recent data showed that uORFs can encode for biologically active proteins and human leukocyte antigen (HLA)-presented peptides in malignant and benign cells suggesting their potential role in cancer cell development and survival. However, the role of uORFs in translational regulation of cancer-associated transcripts as well as in cancer immune surveillance is still incompletely understood.

**Methods:**

We examined the translational regulatory effect of 29 uORFs in 13 cancer-associated genes by dual-luciferase assays. Cellular expression and localization of uORF-encoded peptides (uPeptides) were investigated by immunoblotting and immunofluorescence-based microscopy. Furthermore, we utilized mass spectrometry-based immunopeptidome analyses in an extensive dataset of primary malignant and benign tissue samples for the identification of naturally presented uORF-derived HLA-presented peptides screening for more than 2000 uORFs.

**Results:**

We provide experimental evidence for similarly effective translational regulation of cancer-associated transcripts through uORFs initiated by either canonical AUG codons or by alternative translation initiation sites (aTISs). We further demonstrate frequent cellular expression and reveal occasional specific cellular localization of uORF-derived peptides, suggesting uPeptide-specific biological implications. Immunopeptidome analyses delineated a set of 125 naturally presented uORF-derived HLA-presented peptides. Comparative immunopeptidome profiling of malignant and benign tissue-derived immunopeptidomes identified several tumor-associated uORF-derived HLA ligands capable to induce multifunctional T cell responses.

**Conclusion:**

Our data provide direct evidence for the frequent expression of uPeptides in benign and malignant human tissues, suggesting a potentially widespread function of uPeptides in cancer biology. These findings may inspire novel approaches in direct molecular as well as immunotherapeutic targeting of cancer-associated uORFs and uPeptides.

**Supplementary Information:**

The online version contains supplementary material available at 10.1007/s00018-022-04145-0.

## Background

The development and advances of ribosome profiling [1, 2] has uncovered numerous sites of active translation at upstream open reading frames (uORFs) preceding the main protein-coding sequences (CDS) of eukaryotic transcripts [3, 4]. While approximately 55% of human transcript leader sequences (TLSs) contain canonical upstream AUG (uAUG) initiation codons [5], virtually all human transcripts carry near-cognate alternative translational initiation sites (aTISs), differing in one base from the canonical AUG sequence [6, 7]. Computational and experimental studies demonstrated compelling evidence for an important regulatory role of uORF-mediated translational control in (patho-)physiology [3, 8–10], and several uORF-associated genetic variants have been linked to the development of disease [8, 10–15]. A recent study also demonstrated that virus-derived uORFs are translated during infection and contribute to virulence [16].

Upstream ORFs represent important relays of gene expression regulation, as translation of the downstream CDS from uORF-bearing transcripts requires leaky scanning across the uORF start site or reinitiation of ribosomes after translating the uORF [9, 17, 18]. Upstream ORF-mediated translational regulation has been observed in multiple transcripts across eukaryotic species [8, 19–21]. Specific arrangements of multiple uORFs have been shown to mediate the paradoxical induction of downstream protein translation under conditions of cellular stress, as studied in detail for the transcription factors GCNA4 in yeast, and for ATF4 and ATF5 in mammals [22–25]. Furthermore, several sequencing studies demonstrated frequent genetic variability of uORFs in human cancer [11, 15, 26] and additional individual reports on CDKN1B and CDKN2A directly linked defective uORF-mediated translational control to tumorgenesis [12, 13]. However, only a few reports provided individual experimental evidence for the regulatory impact of uAUG and aTIS uORFs in human proto-oncogenes [11–13, 15, 26, 27].

Very recent studies combining ribosome profiling, proteomics and immunopeptidomics [28–32] confirmed the widespread translation of cryptic peptides from non-coding regions, including 5′-TLSs and 3′-UTRs, non-coding RNAs, intronic, intergenic, and off-frame regions, and provided first insights into their presentation on human leukocyte antigen (HLA) class I molecules. Thereby, uORF-derived peptides encoded in the TLSs of protein-coding transcripts represent the largest category of detected cryptic peptides [30]. Upstream ORF-derived peptides may form direct complexes with their associated main proteins, can act in *cis*- and *trans-*regulatory ways, and may sense the cellular levels of small molecules or metabolites to serve as pepto-switches regulating downstream translation [33–35]. For example, a uPeptide in the TLS of PKC was recently shown to suppress tumor progression, proliferation, invasion and metastasis in different models of breast cancer [36]. Especially in the context of pathologically altered cellular processes such as malignant transformation, the differential translation of uORFs and differential uPeptide processing could produce tumor-specific uORF-derived HLA ligands (HLA uLigands) that may serve as rejection antigens [12, 13, 37]. However, previous immunopeptidomic studies were mainly limited to cell lines and only applied sample-specific proteogenomic approaches using personalized reference databases [29, 32]. Furthermore, cancer-associated HLA presentation was retrospectively determined based on RNA sequencing data [29, 32] due to the lack of complete tissue immunopeptidomics reference libraries from healthy tissues, calling for the direct immunopeptidome analysis of primary malignant and benign tissue samples to further delineate the role of HLA uLigands as tumor-specific targets and their role in anti-tumor immunity.

Here we experimentally characterized the translational regulatory role of selected uORFs in cancer-associated transcripts and present evidence for frequent translation and cellular expression of the related uORF-derived uPeptides. Mass spectrometry-based immunopeptidome analyses using a broadly applicable non-personalized uORF database in an extensive dataset of primary malignant and benign tissue samples further delineated several tumor-associated HLA uLigands capable to induce multifunctional peptide-specific T cell responses.

## Materials and methods

### Selection of uORFs for functional analysis

The selection of uORF containing genes for functional analysis was based on documented oncogenic functions of the associated transcripts [38, 39], high conservation of uORF sequences (PhyloCFS score > 50), or evidence for active uORF translation (TEscore ≥ 5) [4]. This selection yielded a total of 536 genes. Oncogenes were filtered for presence in canonical cancer pathways (RTK/RAS, cell cycle, PI3K, P53, MYC and WNT pathway) where deregulation of a single protein may be sufficient to mediate oncogenic effects on downstream signalling [40]. Then, uORFs of the selected genes were ranked according to the highest uORF score (top 10%) as described in McGillivray* et al.* [3]. This score predicts functional uORF relevance based on specific features, including uORF length, position, and conservation as well as expression of the associated downstream main protein. For each gene, we selected the uAUG with the highest uORF score, the aTIS with the highest uORF score, and the uAUG/aTIS with the highest uPeptide score for experimental analysis. Upstream ORFs < 24 bp were excluded as they were considered to be too small for immunoblot detection. We then determined the presence of the selected uAUGs and aTISs in all RefSeq transcript variants of the respective gene according to the genomic position given in McGillivray *et al.* [3] (hg19) and picked one representative TLS to be used for further experiments. If there were multiple transcript variants including all uORFs under investigation we preferred low complexity TLS as defined by short length, low number of additional uORFs and low number of exons to ease experimental handling. From the remaining set, we finally selected 13 transcripts based on the abundance of previous literature indicating functional oncogenic importance or suggesting active uORF regulation in the respective genes.

### Cell culture

HEK293T cells (obtained from ATCC) were cultivated at 37 °C, 5% (v/v) CO_2_ in humidified and DMEM culture medium supplemented with 10% fetal bovine serum and 1% penicillin/streptomycin.

### Dual-luciferase assay

Complete wt TLSs including the endogenous main ORF (mORF) initiation codon and the Kozak base at position + 4 were synthesized by GeneArt (Thermo Fisher Scientific) (Supplementary Table 1). TLSs were isolated from GeneArt vectors using the appropriate restriction enzymes and were ligated into a translational control reporter plasmid (TCRP) based on the pGL3 basic vector as previously described [5] (Supplementary Figure 1a). Individual uORF initiation codons were mutated to CUC (ΔuORF) wherever possible by site-directed mutagenesis (SDM) or to alternative non-initiation codons (Supplementary Tables 2 and 3). Correctness of all insertions and SDMs was verified by Sanger sequencing. The wt and ΔuORF TCRPs were co-transfected together with a Renilla luciferase control vector (pRL-CMV, Promega) into HEK293T cells using METAFECTENE® transfection reagent (Biontex) according to manufactures instructions. 44 h later cells were washed, lysed, and Firefly and Renilla luciferase activities were measured using a multilabel plate reader (VictorTM X3, PerkinElmer) as described before [41]. In detail, 50,000 HEK293T cells were seeded in 24-well plates, grown for one day and subsequently transfected using TLS-specific amounts of translational reporter plasmid, 75 ng/well Renilla luciferase vector and 3 µl METAFECTENE® mixed in 100 µl Opti-MEM® (Thermo Fisher Scientific). The use of TLS-specific amounts of TCRP (range 1–364.5 ng/well) was required to adjust luminescent signals to the linear range of detection of the plate reader, as individual wt TLS caused largely diverging global inhibitory effects on luciferase expression (Supplementary Figure 2). After 15 min of incubation at room temperature, 50 µl of transfection mix was added drop-wise to the cells in duplicates. 44 h later cells were washed with 500 µl PBS and then lysed using Luciferase Lysis Buffer (90 mM K_2_HPO_4,_ 9 mM KH_2_PO_4_, 0.2% Triton X-100) containing 40 µl Proteinase-Inhibitor Cocktail Complete (Sigma Aldrich). For complete lysis cells were shaken on ice for 30 min. Cell lysates were transferred to a 1.5 ml tube and centrifuged at 21,000*g* at 4 °C for 10 min. The supernatant was transferred in a new 1.5 ml tube and triplicate measurements of each lysate were performed in a NuncTM F96 MicroWellTM polystyrol plate (Thermo Fisher Scientific).

### Real-time quantitative PCR (RT-qPCR)

Whole RNA was isolated from washed and pelleted cells using the Nucleo Spin® RNA Kit (Macherey–Nagel) including a first DNAseI digestion step according to the manufacturer′s instructions. Afterwards, a second DNAseI treatment was performed with 1 µg of isolated RNA. cDNA was synthesised from 200 ng RNA according to the protocol of the RevertAid Strand cDNA Synthesis Kit (Thermo Fisher Scientific) and the final cDNA concentration was adjusted to 100 ng/µl. Relative real-time quantitative PCR (RT-qPCR) was performed in a MicroAmp® Fast 96-well Reaction Plate (0.1 ml) (Thermo Fisher Scientific) using the Luna® Universal qPCR Mastermix (NEB) and following primers: Firefly_for ATCCATCTTGCTCCAACACC, Firefly_rev TCGCGGTTGTTACTTGACTG, Renilla_for GGAATTATAATGCTTATCTACGTGC, Renilla_rev CTTGCGAAAAATGAAGACCTTTTAC. RT-qPCR was performed in a StepOnePlus Real-Time PCR System (Applied Biosystems). To exclude relevant plasmid DNA contamination in RNA extracts, we always included RNA control samples without reverse transcription in RT-qPCR experiments.

### Detection of HA-tagged uPeptides by immunoblotting

Oligonucleotides including the 3xHA-Tag sequence were annealed and ligated into the pcDNA3.1(+) vector using BamHI and XbaI restriction sites. Next, complete TLSs sequences including the uORFs under investigation were amplified by PCR from Firefly luciferase vectors deleting the uORF’s termination codon and including HindIII and BamHI restriction site-overhangs for ligation. Amplicons were ligated upstream of the 3xHA-tag into the pcDNA3.1(+)-3xHA vector with the uPeptide initiation codon being in-frame with the 3xHA-tag (Supplementary Figure 1b). We generated expression vectors for all AUG uORFs, all uORFs with the highest uPeptides scores, and additionally included the CTNNB1 aTIS uORF, as this gene lacked an AUG uORF but contained a UUG.1 aTIS uORF with the highest uORF score on a distinct transcript variant. Correctness of insertions was verified by Sanger sequencing. 500,000 HEK293T cells were seeded in a 6-well plate and grown for 24 h. 3 µg of expression vector was transfected using 5 µl METAFECTENE® (Biontex). After 44 h cells were treated with 2 µl of 10 µM MG132 (Enzo life sciences) and 8 h later cell lysates were subjected to immunoblotting following standardized protocols using Vinculin (7F9) (sc-73614) and HA (F-7) (sc-7392) antibodies (Santa Cruz). In detail, cells were washed with 1 ml PBS and lyzed with 150 µl Immunoblot Lysis buffer (50 mM Tris pH 7.4, 150 mM NaCl, 0.1% Triton X-100, 1 mM EDTA) containing 6 µl Proteinase-Inhibitor Cocktail Complete (Sigma–Aldrich) and 1.5 µl DTT by shaking for 30 min at 4 °C. Lysates were centrifuged for 20 min by 21,000×*g* at 4 °C and supernatants were transferred to a new 1.5 ml Eppendorf tube. Total protein amounts were determined using the BCA assay. 5 µl of NuPage® LDS Sample Buffer (4x) (Invitrogen) was added to 20 µl protein lysate containing 50 µg of total protein. The mixture was incubated at 9 °C for 5 min and subsequently applied on a 22% SDS-Page gel. After electrophoretic separation proteins were transferred on a PVDF membrane using the Mini Trans-Blot® cell at 100 V for 1.5 h. After blocking the membrane in 5% skim milk for 1 h, the upper part (> 100 kDa) was incubated with Vinculin antibody (1:5,000 in 5% skim milk) and the lower part (< 100 kDa) with the HA antibody (1:1,000 in 5% skim milk) at 4 °C over night. Membrane was washed with 1 × TBST three times for 5 min and then incubated with goat anti-mouse antibody (1:5,000 in 5% skim milk, Jackson Immuno Research AB_2338461) for 1 h. The membrane was again washed three times in 1 × TBST for 5 min and the Super Signal™ West Pico PLUS Chemiluminescent Substrate (Thermo Fisher Scientific) was added to the membrane according to the manufacturer’s protocol. Immunoblots were developed using the Amersham Imager 600 (GE Healthcare). Exposure times were one minute for the immunoblots showing ASNSD1, ATF5, MAPK1, and MDM2 uPeptides, 4 min for the immunoblot of the CTNNB1 uPeptide and 3.5 min for the immunoblot of the TMEM203 uPeptide.

### Immunofluorescence-based microscopy

For each uORF individual TLSs including the sequence from the 5′-cap to the disrupted termination codon of the uORF under investigation were isolated from the pcDNA3.1( +)-3xHA vector using the restriction enzymes HindIII and BamHI and were ligated upstream and in-frame to the EGFP coding sequence (with deleted EGFP-initiation codon) into the pEGFP N3 vector (Addgene) and correct insertion was verified by Sanger sequencing (Supplementary Figure 1c). 200,000 HEK293T cells were seeded on a microscope cover-glass in a 12-well plate. After 24 h cells were transfected using METAFECTENE® as described above. After another 24 h the cells were washed with cold PBS, permeabilized by a 5 min treatment with 100 µl Methanol (−20 °C) and fixed in 4% paraformaldehyde for 10 min, and washed again with cold PBS. Cover-glasses were then treated with 30 µl DAPI-containing mounting medium (Thermo Fisher Scientific) and placed in the middle of a microscopy object slide. Image acquisition, analysis, and processing were carried out using a Leica SP8 FLIM Microscope and ImageJ software [42]. Here, the captured z-stack images are presented as merged z-stack images.

### Sample collection for immunopeptidomic analysis

For immunopeptidome analysis, peripheral blood mononuclear cells (PBMCs) or bone marrow mononuclear cells (BMNCs) from acute myeloid leukemia (AML) and chronic lymphocytic leukemia (CLL) patients were collected at the Department of Hematology and Oncology at the University Hospital Tübingen, Germany. Samples of CLL patients (*n* = 15) were collected at the time of first therapy indication according to iwCLL guidelines [43]. Samples of AML patients (*n* = 15) were collected at the time of diagnosis (*n* = 13), under palliative therapy (*n* = 1) or at relapse (*n* = 1). PBMCs from healthy volunteers (HVs) and CD34^+^ magnetically enriched hematopoietic progenitor cells (HPCs, CD34 MicroBead Kit, human, Miltenyi Biotec) from hematopoietic stem cell aphereses from G-CSF mobilized blood donations of HVs and patients with non-hematological malignancies (*e.g.* germ cell tumors) were collected at the University Hospital Tübingen, Germany. Cells were isolated by density gradient centrifugation and stored at −80 °C until further use. Informed consent was obtained in accordance with the Declaration of Helsinki protocol. The study was performed according to the guidelines of the local ethics committees (373/2011B02, 454/2016B02, 406/2019B02). HLA typing was carried out by the Department of Hematology and Oncology, Tübingen, Germany. Furthermore, we used two publically available immunopeptidomic datasets comprising samples of ovarian carcinoma (OvCa) and benign ovaries (OvN) [44] as well as melanoma (Mel) [45]. Sample characteristics of malignant and benign tissue samples are provided in Supplementary Tables 4 and 5, respectively.

### Isolation of HLA ligands

HLA class I molecules were isolated by standard immunoaffinity purification as described before [47] using the pan-HLA class I-specific W6/32 monoclonal antibody (produced in-house).

### Analysis of HLA ligands by liquid chromatography-coupled tandem mass spectrometry (LC–MS/MS)

HLA ligand extracts were analyzed as described previously [47, 48]. Peptides were separated by nanoflow high-performance liquid chromatography (RSLCnano, Thermo Fisher Scientific) using a 50 μm × 25 cm PepMap rapid separation liquid chromatography column (Thermo Fisher Scientific) and a gradient ranging from 2.4% to 32.0% acetonitrile over the course of 90 min. Eluted peptides were analyzed in an online-coupled LTQ Orbitrap XL or LTQ Orbitrap Fusion Lumos mass spectrometer (Thermo Fisher Scientific) equipped with nano-electronspray ion sources using a data-dependent acquisition mode employing a top five or a top speed collision-induced dissociation (CID) fragmentation method (normalized collision energy 35%), respectively. The mass range was set to 400–650 m/z with charge states 2+ and 3+ selected for fragmentation.

### Data processing, uORF database structure, and HLA annotation

For data processing, the software Proteome Discoverer (v1.4.0, Thermo Fisher) was used to integrate the search results of the SEQUEST HT search engine (University of Washington) [49] against the human proteome as comprised in the Swiss-Prot database (20,367 reviewed protein sequences, January 7th 2020) supplemented with two datasets of uORF sequences. The datasets of uORF sequences contained 1062 uORFs (877 different amino acid sequences) with the highest scores predicting uORF functionality (McGillivray set [3]) and 1236 uORFs (1235 different amino acid sequences with five sequences contained in both sets) selected based on indications of functional relevance from previous experimental data, genetic context or sequence analysis (in-house set). No enzymatic restriction was applied. Precursor mass tolerance was set to 5 ppm, and fragment mass tolerance to 0.5 Da for ion trap spectra and 0.02 Da for orbitrap spectra, respectively. Oxidized methionine was allowed as a dynamic modification. The false discovery rate (FDR) was estimated using the Percolator algorithm (v2.04) [50] and limited to 5%. Peptide lengths were limited to 8–12 amino acids. Protein inference was disabled, allowing for multiple protein annotations of peptides. HLA class I annotation was performed using NetMHCpan 4.0 [51–53] and SYFPEITHI 1.0 [54] annotating peptides with percentile rank below 2% and ≥ 60% of the maximal score, respectively. We screened the immunopeptidomes for uORF-derived peptide sequences, which are uniquely mapped on uORF sequences and not on any other non-uORF human protein sequence (expect for ASDURF_HUMAN, a reviewed uORF).

### Peptide synthesis

Peptides were produced by the peptide synthesizer Liberty Blue (CEM) using the 9-fluorenylmethyl-oxycarbonyl/tert-butyl strategy [55].

### Spectrum validation

Spectrum validation of the experimentally eluted peptides was performed by computing the similarity of the spectra with corresponding isotope-labeled synthetic peptides measured in a complex matrix. The spectral correlation was calculated between eluted peptide spectra and synthetic peptide spectra using the intensities of annotated b- and y-ion peaks. For synthetic peptide-based validation of mass spectrometry-based peptide identifications a panel of 18 tumor-associated, tumor-enriched, high frequent, and reviewed HLA uLigands was selected.

### Blood samples for T cell-based assays

PBMCs from whole blood samples of HVs were isolated by standard density gradient centrifugation and CD8^+^ T cells were magnetically isolated (CD8 MicroBeads, human, Miltenyi Biotec). Blood samples were kindly provided by the Institute for Clinical and Experimental Transfusion Medicine at the University Hospital Tübingen after obtaining written informed consent.

### Refolding

Biotinylated HLA-peptide complexes were manufactured as described previously [56] and tetramerized using PE-conjugated streptavidin (Invitrogen Life Technologies) at a 4:1 molar ratio.

### Induction of peptide-specific CD8^+^ T cells with artificial antigen-presenting cells (aAPC)

Priming of peptide-specific cytotoxic T lymphocytes was conducted using artificial antigen-presenting cells (aAPCs) as described previously [57]. In detail, 800,000 streptavidin-coated microspheres were loaded with 200 ng biotinylated HLA:peptide monomer and 600 ng biotinylated anti-human CD28 monoclonal antibody (mAb, clone 9.3, in-house production). CD8^+^ T cells were cultured with 4.8 U/µl IL-2 (R + D) and 1.25 ng/ml IL-7 (PromoKine). Weekly stimulation with aAPCs (200,000 aAPCs per 1 × 10^6^ CD8^+^ T cells) and 5 ng/ml IL-12 (PromoKine) was performed four times.

### Cytokine and tetramer staining

The frequency and functionality of peptide-specific CD8^+^ T cells was analyzed by tetramer [58] and intracellular cytokine staining (ICS) [59, 60], respectively, as described previously. For ICS, cells were pulsed with 10 μg/ml of individual peptide and incubated with 10 μg/ml Brefeldin A (Sigma–Aldrich) and 10 μg/ml GolgiStop (BD) for 12–16 h. Staining was performed using Cytofix/Cytoperm (BD), PerCP anti-human CD8, PacificBlue anti-human TNF, FITC anti-human CD107a (BioLegend), and PE antihuman IFN-γ antibodies (BD). PMA and ionomycin (Sigma-Aldrich) served as a positive control. The peptides YLLPAIVHI (HLA-A*02, DDX5_HUMAN_148-156_), RLRPGGKKK (HLA-A*03, GAG_HV1BR_20-28_), and TPGPGVRYPL (HLA-B*07, NEF_HV1BR_128-137_) served as negative control peptides. The frequency of peptide-specific CD8^+^ T cells after aAPC-based priming was determined by tetramer staining using PerCP anti-human CD8 antibody and HLA:peptide tetramer-PE. For negative control, tetramers of the same HLA allotype containing irrelevant control peptides were used. The priming was considered successful if the frequency of peptide-specific CD8^+^ T cells was > 0.1% of CD8^+^ T cells within the viable single-cell population and at least three-fold higher than the frequency of peptide-specific CD8^+^ T cells in the negative control. The same evaluation criteria were applied for the ICS results. All samples were analyzed on a FACS Canto II cytometer (BD).

### Software and statistical analysis

Overlap analysis was performed using BioVenn [61]. The population coverage of HLA allotypes was calculated by the IEDB population coverage tool (www.iedb.org) [62, 63]. Fisher’s exact test was used for the analysis of HLA allotype distribution between the immunopeptidome dataset (*n* = 90), the world population (*n* = 90,046) and the European population (*n* = 32,856) [64] as well as between the malignant (*n* = 45) and benign (*n* = 45) tissue dataset. Flow cytometric data were analyzed using FlowJo 10.0.8 (Treestar). All figures were generated using GraphPad Prism 9.0.2 (GraphPad Software).

## Results

### Upstream ORF-mediated translational regulation of CDS expression in cancer-associated transcripts

**Fig. 1:**
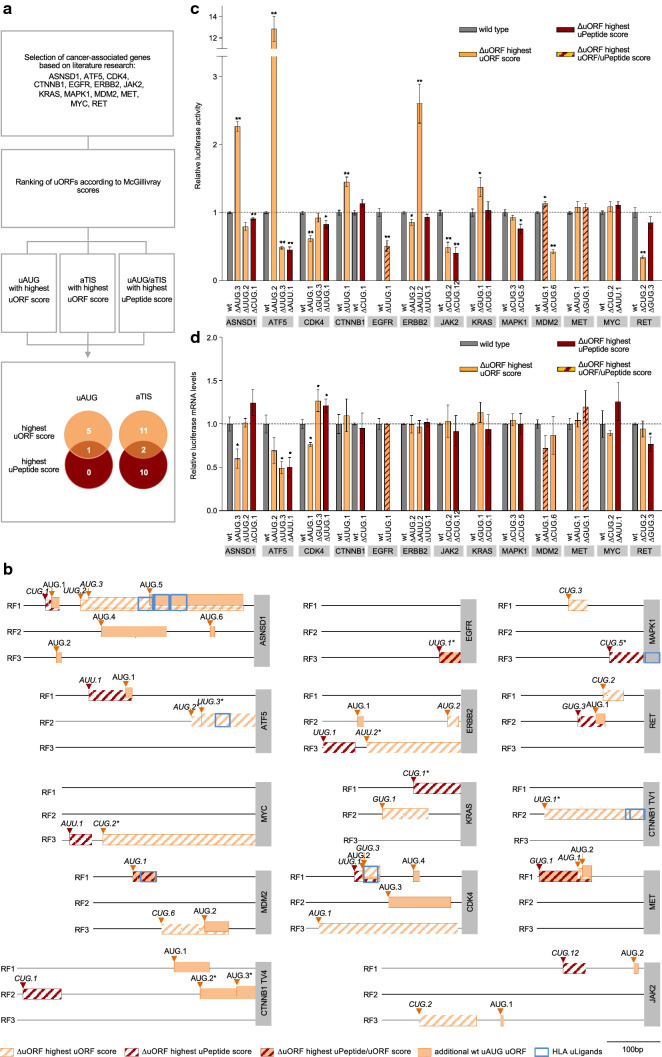
Translational regulation of CDS expression through uORFs in cancer-associated transcripts.** a** Flowchart illustrating the selection process for uORFs initiated at canonical (uAUG) and at alternative translational initiation sites (aTIS) for functional analysis.** b** Schematic illustrations of indicated in-scale TLSs displaying the analyzed uORFs (stripped boxes) and additional wild type AUG uORFs (wt, filled orange boxes) on reading frames (RF) 1–3 (black lines). Upstream ORFs overlapping into the CDS are marked with an asterisk at the respective start codon. Grey boxes on the right contain gene symbols and indicate the start of the CDS. Note that for CTNNB1 the uORFs with the highest uORF- and uPeptid-scores mapped to distinct transcript variants. Accordingly, both were selected for experimental analysis. **c** Bar graphs showing relative Firefly luciferase activities detected for indicated wild type (wt) and ΔuORF TLSs normalized to Renilla luciferase internal controls. Results are combined from three independent experiments and error bars indicate SEM. Levels of significance are *p* ≤ 0.05 (*) and *p* ≤ 0.01 (**) as determined by two-tailed nonparametric Mann–Whitney *U*-tests. **d** Bar graphs indicating relative Firefly luciferase mRNA levels for indicated wt and ΔuORF TLSs normalized to Renilla luciferase internal control. Results are combined from three independent experiments and error bars indicate SEM. Levels of significance are *p* ≤ 0.05 (*) and *p* ≤ 0.01 (**) as determined by two-tailed nonparametric Mann–Whitney *U*-tests

Aiming to characterize the functional impact of uORF-mediated translational regulation and the prevalence of uPeptide expression in cancer-related genes, we first selected a set of 29 uAUG and aTIS uORFs from 13 cancer-associated genes (Fig. 1a, Supplementary Table 3). Upstream ORFs were selected based on literature research [4, 38, 39] and categorized according to the type of initiation codon (uAUG vs. aTIS) and the computationally defined uORF and uPeptide scores [3] predicting functional relevance. All candidate uORFs were tested for their translational regulatory impact on downstream CDS translation in dual-luciferase reporter assays [41] using wild type (wt) TLSs and TLSs carrying a functionally deleted uORF initiation codon (ΔuORF) (Supplementary Figure 1a).

Structurally, individual TLSs showed high variability with respect to TLS length, as well as uORF number, length and position (Fig. 1b), resulting in variable levels of baseline relative luciferase activity for individual wt TLSs (Supplementary Figure 2). The functional ablation of uAUG and aTIS initiation codons caused significant changes of luciferase activity for 18 of 29 uORFs, ranging from 12.83-fold induction to 0.34-fold repression of luciferase signals as compared to wt TLSs (Fig. 1c). In 15 of these cases, concomitant monitoring of luciferase mRNA levels excluded major contributions of alterations in luciferase transcript levels, suggesting a mostly translational regulatory effect of the ΔuORF variants (Fig. 1d). Six of these uORF ablations induced a ≥ 2-fold increase or ≤ 0.5-fold decrease of luciferase activity, respectively. Most prominent induction of relative luciferase activity was detected for the AUG.2 > CUC ΔuORF TLS of the activating transcription factor 5 (ATF5, 12.83 ± 1.64 SEM, *p* ≤ 0.01), the AUU.2 > CUC ΔuORF TLS of receptor tyrosine kinase Erb-B2 (ERBB2, 2.60 ± 0.63 SEM, *p* ≤ 0.01), and the AUG.3 > CUA ΔuORF TLS of Asparagin synthetase domain-containing protein 1 (ASNSD1, 2.27 ± 0.10 SEM, *p* ≤ 0.01). Major reduction of relative luciferase activity compared to wt TLS levels was observed for the CUG.2 > CUC ΔuORF TLS of the receptor tyrosine kinase Ret (RET, 0.34 ± 0.03 SEM, *p* ≤ 0.01), the CUG.12 > CUC and the CUG.2 > CUC ΔuORF TLS of janus kinase 2 (JAK2, 0.41 ± 0.11 SEM, *p* ≤ 0.01 and 0.49 ± 0.12, *p* ≤ 0.01, respectively), and the CUG.6 > CUC ΔuORF-TLS of the murine double minute 2 homolog (MDM2, 0.53 ± 0.09 SEM, *p* ≤ 0.01). Overall, a translational regulatory effect was observed for 4 of 6 uAUG and 11 of 23 aTIS uORFs, suggesting that both, canonical uAUG and aTIS initiation codons may be similar functionally relevant for the regulation of cancer-associated gene expression and may impact cancer onset and progression.

### Translation and cellular localization of uPeptides

**Fig. 2:**
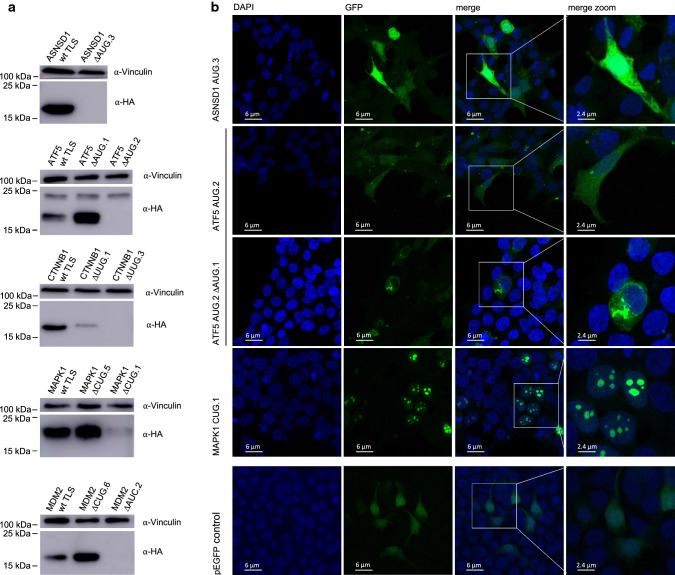
Evidence for translation and specific cellular localization of uPeptides in cancer-associated transcripts. **a** Representative immunoblots of ≥ 3 independent experiments using HEK293T cell lysates prepared 52 h after transfection of expression vectors containing indicated triple HA-tagged wt and ΔuORF TLS variants. Eight hours prior to lysis cells were exposed to proteasome inhibitor MG132. **b** Representative pictures of ≥ 3 independent experiments showing the expression and intracellular localization of indicated EGFP-tagged uPeptides as detected 24 h after transfection of HEK293T cells. Upstream peptides were expressed from TLS-EGFP-expression vectors containing the complete 5′-upstream sequence of indicated wt TLSs and an EGFP-tag replacing the uStop codon of the investigated uORF. The pictures shown here are presented as merged z-stack images. Additional pictures are presented in Supplementary Fig. 5

Focussing on AUG uORFs and uORFs with the highest uPeptide scores, we analyzed whether uORFs encoded by cancer-associated transcripts were translated into uPeptides in vitro. Five of 19 HA-tagged uPeptides, translated from the TLSs of ASNSD1, ATF5, beta-Catenin (CTNNB1), mitogen-activated protein kinase 1 (MAPK1), and MDM2, were detected by immunoblotting (Fig. 2a). Introduction of start codon ablating AUG.3 > CUC and AUG.2 > CUC mutations into the ASNSD1 and ATF5 TLSs resulted in complete losses of the uPeptide bands (Fig. 2a), validating translational initiation at the computationally predicted uORF start codons. To identify the origin of the larger 23 kDa band in the ATF5 immunoblots, we introduced several regional and codon-specific mutations to the ATF5 TLS (Supplementary Figure 3). The data indicated that this band represented an extended ATF5 uPeptide initiated by an obscure start site differing from classical uAUG and aTIS codons. As the functional deletions of the predicted uORF initiation codons did not always result in complete ablation of the HA-tagged uPeptides (Fig. 2a), we next inserted a number of additional uStart deleting mutations into the CTNNB1, MAPK1 and MDM2 TLSs (Supplementary Table 6). For CTNNB1, expression of the uPeptide was markedly reduced upon deletion of the predicted UUG.1 uORF start site and was undetectable upon insertion of a UUG.3 > UCG mutation. In the case of MAPK1, the deletion of the predicted CUG.5 codon had no effect on uPeptide expression in immunoblot analysis, while an alternative CUG.1 > CGC mutation strongly reduced MAPK1 uPeptide expression as compared to wt levels (Fig. 2a). Similarly, deletion of the predicted CUG.6 uORF start codon in the TLS of MDM2 did not abolish uPeptide expression, but the uPeptide signal was lost upon insertion of an AUC.2 > ACC mutation (Fig. 2a). Of note, mutational ablation of an AUG.2 codon immediately upstream of the AUC.2 codon had no detectable effect on the MDM2 uPeptide expression (Supplementary Figure 4).

Aiming to validate uPeptide expression by an independent experimental approach, we performed immunofluorescence-based microscopy of EGFP-labeled uPeptides. The ASNSD1 and ATF5 uPeptides showed ubiquitous and predominantly cytosolic cellular localization, respectively (Fig. 2b, Supplementary Figure 5). Interestingly, an AUG.1 > CUC deletion of the ATF5 AUG.1 uORF not only induced higher ATF5 AUG.2 uPeptide levels in immunoblot analyses (Fig. 2a), but was also associated with a marked change in cellular localization of the AUG.2 uPeptide, now frequently accumulating in perinuclear focal structures (Fig. 2b, Supplementary Figure 5). The MAPK1 CUG.1 peptide also showed specific focal localization, exclusively mapping to the nucleus and implying a potential functional relevance for MAPK1 signalling (Fig. 2b, Supplementary Figure 5). Together, these data confirmed the cellular expression of uPeptides from cancer-associated transcripts and demonstrated uPeptide-specific cellular localizations, suggesting distinct functions of individual uPeptides as *trans*-acting factors.

### Mass spectrometry-based immunopeptidome analysis identified naturally presented HLA uLigands

**Fig. 3:**
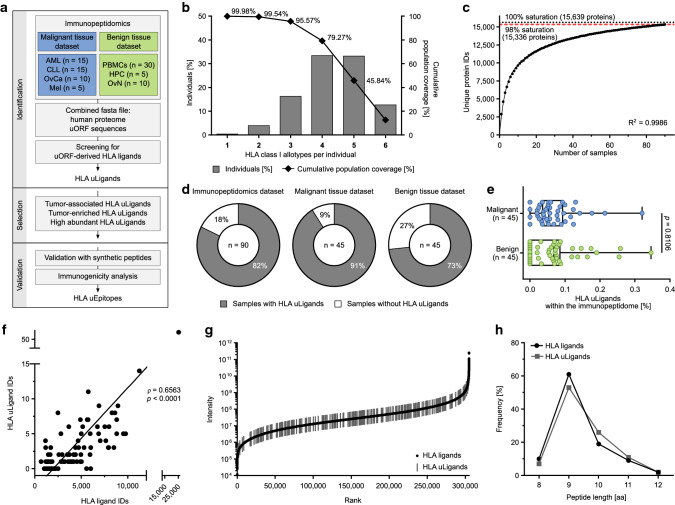
Mass spectrometry-based identification of uORF-derived HLA ligands. **a** Workflow of immunopeptidomics-based identification of uORF-derived HLA ligands (HLA uLigands) and T cell epitopes (HLA uEpitopes). **b** HLA class I allotype population coverage within the immunopeptidomics dataset (*n* = 90) compared to the world population (calculated by the IEDB population coverage tool, www.iedb.org). The frequencies of individuals within the world population carrying up to six HLA allotypes (*x*-axis) included in the immunopeptidomics dataset are indicated as grey bars on the left *y*-axis. The cumulative percentage of population coverage is depicted as black dots on the right *y*-axis. **c** Saturation analysis of HLA ligand source proteins of the immunopeptidomics dataset (*n* = 90). Number of unique HLA ligand source protein identifications shown as a function of cumulative immunopeptidome analysis. Exponential regression allowed for the robust calculation (*R*^2^ = 0.9986) of the maximum attainable number of different source protein identifications (100% saturation, dotted line). The dashed red line depicts the source proteome coverage achieved in the immunopeptidome dataset. **d** Pie charts depicting the percentage of samples with identified HLA uLigands within the total immunopeptidomics dataset comprising malignant and benign tissue samples (*n* = 90, left panel) as well as within the malignant (*n* = 45, middle panel) and benign (*n* = 45, right panel) tissue datasets separately. **e** Percentage of HLA uLigands within the immunopeptidome of malignant and benign tissue samples (boxes represent median and 25th–75th percentiles, whiskers are minimum to maximum, two-sided Mann–Whitney *U*-test). **f** Correlation of total HLA ligand identifications with HLA uLigand identifications in the immunopeptidome dataset (*n* = 90). Dots represent individual samples. Spearman’s rho (*ρ*) and *p*-value. **g** Ranked intensity values of mass spectrometry-acquired data derived from the combined immunopeptidomes of all samples (*n* = 90). Positions of HLA uLigands are projected on the curve. **h** Peptide length distribution of HLA uLigands and all identified HLA ligands. *AML* acute myeloid leukemia, *CLL* chronic lymphocytic leukemia, *OvCa* ovarian carcinoma, *Mel* melanoma, *PBMCs* peripheral blood mononuclear cells, *HPC* CD34-enriched hematopoietic progenitor cells, *OvN* benign ovaries, *HLA uLigands* uORF-derived HLA ligands, *IDs* identifications, *aa *amino acid

To evaluate if uORFs encode HLA-presented peptides that might serve as antigenic targets for cancer immune surveillance and immunotherapeutic approaches, mass spectrometry-based immunopeptidome profiling of primary malignant [*n* = 45, AML (*n* = 15), CLL (*n* = 15), OvCa [44] (*n* = 10), and Mel [45] (*n* = 5)] as well as benign tissue samples [*n* = 45, PBMCs (*n* = 30), CD34-enriched HPC (*n* = 5), and OvN (*n* = 10)] was applied (Fig. 3a). This immunopeptidomic dataset covers 49 different HLA class I allotypes including 14 different HLA-A, 22 HLA-B, and 13 HLA-C allotypes. HLA allotype frequencies are comparable to the world and European population with 96% of the allotypes showing no significant differences in the frequency between the immunopeptidome dataset compared to the world and the European population (Supplementary Figure 6a). Between the malignant and benign tissue datasets comparable HLA allotype frequencies are observed for 96% of the allotypes (Supplementary Figure 6b). 99.98% of the world population carries at least one of the HLA class I allotypes included in the dataset (Fig. 3b). We identified a total of 127,766 unique HLA class I ligands (peptides assigned to their HLA allotype, range 684–25,249, mean 4368 per sample, Supplementary Tables 4 and 5) with a FDR of 5% from 15,336 different source proteins, obtaining 98% (97% and 94% for malignant and benign samples, respectively) of the estimated maximum attainable coverage in HLA ligand source proteins (Fig. 3c and Supplementary Figure 7). For the identification of naturally presented HLA uLigands we screened the immunopeptidomes for HLA ligands derived from 1062 uORFs with highest scores predicting uORF functionality [3] and 1236 uORFs manually selected due to previous experimental data [9], genetic context or cancer association (database available at PRIDE PXD025716). Strikingly, HLA uLigands were identified in 82% (74/90) of the samples (91% (41/45) of malignant and 73% (33/45) of benign tissue samples, Fig. 3d). A total of 125 unique HLA uLigands derived from 120 different uORFs of 79 different genes including ASNSD1, ATF5, MAPK1, and transmembrane protein 203 (TMEM203) were identified (Supplementary Data 1). The frequency of HLA uLigands within the total immunopeptidome varies from 0.00 to 0.35% (median 0.06%) with no significant differences between malignant (range 0.00–0.32%, median 0.05%) and benign (range 0.00–0.35%, median 0.07%) tissue samples (Fig. 3e, Supplementary Data 1). The number of identified HLA uLigands correlates significantly with the size of the individual immunopeptidomes (Fig. 3f). HLA uLigands are presented by 30 different HLA class I allotypes (7 HLA-A, 17 HLA-B, 6 HLA-C) with 14/125 HLA uLigands presented on more than one allotype resulting in 140 unique HLA uLigand-allotype combinations (Supplementary Data 1). HLA uLigands showed different ligand- and sample-specific intensity ranks covering the whole range of immunopeptidome peptide abundance (Fig. 3g and Supplementary Figure 8). The peptide length distribution is similar between uORF-derived and non-uORF-derived HLA ligands with 79% and 80% of the HLA ligands being 9/10mers, respectively, showing the characteristic length distribution of HLA class I-presented peptides (Fig. 3h).

### Comparative immunopeptidome profiling delineates tumor-associated and high abundant HLA uLigand presentation in malignancy

**Fig. 4:**
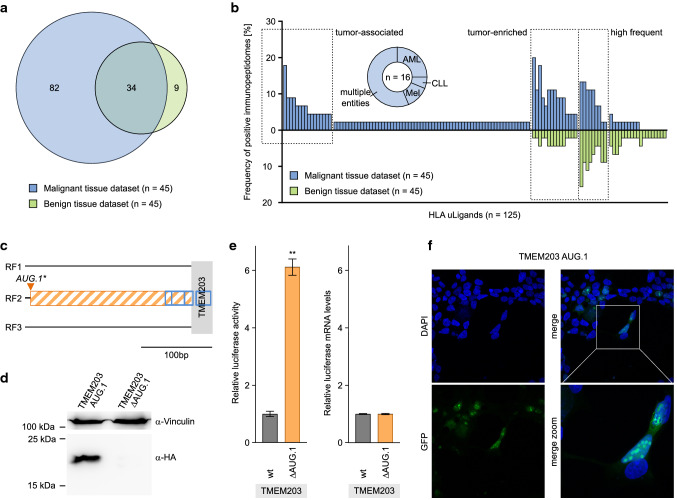
Comparative immunopeptidome profiling identified uORF-derived tumor antigens. **a** Overlap analysis of uORF-derived HLA ligand identifications of primary malignant (*n* = 45) and benign (*n* = 45) tissue samples. **b** Comparative profiling of HLA uLigands (*n* = 125) based on HLA-restricted presentation frequency in malignant and benign immunopeptidomes. Frequencies of positive immunopeptidomes for the respective HLA uLigands (*x*-axis) are indicated on the *y*-axis. The left box highlights tumor-associated antigens (*n* = 16) showing malignant-exclusive frequent presentation. The donut chart displays the entity specificity of tumor-associated HLA uLigands. The middle box marks tumor-enriched antigens displaying two-fold representation frequency on malignant compared to benign tissue samples. The right box highlights high abundant antigens (*n* = 9) showing a frequent presentation in ≥ 5 samples. **c** Schematic illustration of the TMEM203 TLS displaying the only canonical uORF (hatched orange box) on reading frame 2 (black line). The grey box indicates the start of the CDS. Blue open boxes mark the identified HLA uLigands. **d** Representative immunoblot of ≥ 3 independent experiments using HEK293T cell lysates prepared 52 h after transfection of expression vectors containing 3xHA-tagged TMEM203 AUG.1 wt and ΔuORF-TLSs. Eight hours prior to lysis the cells were exposed to proteasome inhibitor MG132. Exposure time was 3.5 min. **e** Bar graph showing relative Firefly luciferase activities and mRNA levels detected for indicated wt and ΔuORF TMEM203 TLSs normalized to Renilla luciferase internal controls. Results are combined from three independent experiments. Error bars indicate SEM. Level of significance *p* ≤ 0.01 (**) as determined by two-tailed nonparametric Mann–Whitney *U*-tests. **f** Representative pictures of ≥ 3 independent experiments showing the intracellular localization of the EGFP-tagged TMEM203 AUG.1 uPeptide as detected 24 h after transfection of HEK293T cells. *AML* acute myeloid leukemia, *CLL* chronic lymphocytic leukemia, *Mel* melanoma, *RF* reading frame, *wt* wild type

For the identification of tumor-associated HLA uLigands, we performed comparative immunopeptidome profiling of the malignant and benign tissue datasets. Overlap analysis of all identified HLA uLigands revealed 66% (82/125) tumor-exclusive HLA uLigands that were never detected on benign tissue samples (Fig. 4a, Supplementary Data 1). For the identification of high frequent tumor-associated uORF antigens, tumor-exclusive HLA uLigands were ranked according to their frequency within the malignant tissue dataset (Fig. 4b). We identified 16/82 (20%) HLA uLigands with representation in two or more malignant tissue samples independent of the HLA allotype. The allotype-specific frequencies within the malignant dataset rose up to 80% for HLA-A*68-, 29% for HLA-A*03-, and 21% for HLA-B*07-restricted HLA uLigands in HLA-matched samples. The 16 tumor-associated uORF antigens could be further divided into tumor entity-specific subgroups with 4/16 AML-specific, 1/16 CLL-specific, and 2/16 Mel-specific HLA uLigands as well as 9/16 HLA uLigands presented by multiple entities (Fig. 4b, Supplementary Data 1). Furthermore, 15/82 (18%) tumor-enriched HLA uLigands defined by at least two-fold higher frequency in the malignant tissue dataset compared to the benign tissue dataset were identified (Fig. 4b, Supplementary Data 1). As a third interesting group of HLA uLigands, high frequent (identified in ≥ 5 samples in the total immunopeptidomics dataset) HLA uLigands (9/125, 7%) presented on both, malignant and benign tissue samples, were distinguished with allotype-specific representation frequencies up to 88% in allotype-matched samples (Fig. 4b, Supplementary Data 1). Since the HLA uLigand P_TMEM203_B*07/C*16_ (RSAGPRPAL) showed the highest frequency of tumor-specific presentation (8/45, 17.8%), we performed additional functional testing on the TMEM203 TLS (Fig. 4c). We detected a strong signal of HA-tagged TMEM203 AUG.1 peptide ectopically expressed in HEK293T cells that was lost upon introduction of an AUG.1 > ACC mutation to the TMEM203 TLS (Fig. 4d). In dual-luciferase reporter assays, we observed a 6.12-fold (± 0.32 SEM, *p* ≤ 0.01) increase of luciferase activity for an AUG.1 > ACC ΔuORF TLS variant compared to wt TLS signals (Fig. 4e). Furthermore, in immunofluorescence experiments the TMEM203 AUG.1 uPeptide localized to the nucleus in the majority of cells, resembling the focal enrichment observed for the MAPK1 CUG.1 uPeptide before. Additionally, in approximately 20% of analyzed EGFP^+^ cells, a focal localization was also observed within the cytoplasm, indicating two potential sites of functional implication (Fig. 4f, Supplementary Figure 5).

### Naturally presented HLA uLigands induce multifunctional peptide-specific T cells

**Fig. 5:**
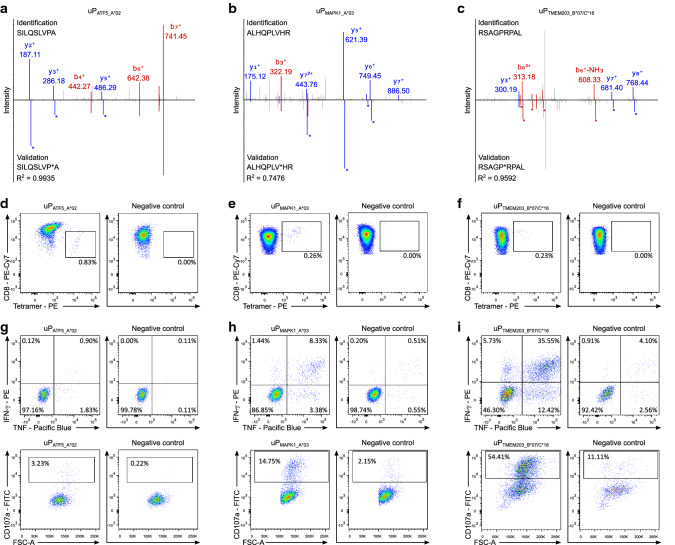
Spectral validation and immunogenicity analyses of uORF-derived tumor antigens. **a**–**c** Validation of the experimentally eluted peptides **a** uP_ATF5_A*02_, **b** uP_MAPK1_A*03_, and **c** uP_TMEM203_B*07/C*16_. Comparison of fragment spectra (*m*/*z* on *x*-axis) of HLA uLigands eluted from primary samples (identification) to their corresponding isotope-labeled synthetic peptides (validation, mirrored on the *x*-axis) with the calculated spectral correlation coefficient (*R*^2^). Identified b- and y-ions are marked in red and blue, respectively. Ions containing isotope-labeled amino acids are marked with asterisks. **d**–**f** Naïve CD8^+^ T cells were primed in vitro using HLA uLigand-loaded aAPCs with the HLA-A*02-, -A*03-, and -B*07-restricted peptides **d** uP_ATF5_A*02_, **e** uP_MAPK1_A*03_, and **f** uP_TMEM203_B*07/C*16_, respectively. Graphs show single, viable cells stained for CD8 and PE-conjugated multimers of indicated specificity. The left panels show HLA uLigand tetramer staining, the right panels (negative control) depict tetramer staining of T cells from the same donor primed with an HLA-matched control peptide. **g**–**i** Functional characterization of HLA uLigand-specific CD8^+^ T cells by intracellular cytokine staining. Representative examples of IFN-γ and TNF production (upper panels) as well as CD107a expression (lower panels) after stimulation of **g** uP_ATF5_A*02_-, **h** uP_MAPK1_A*03_-, and **i** uP_TMEM203_B*07/C*16_-specific CD8^+^ T cells with the HLA-A*02-, -A*03-, and -B*07-restricted peptides uP_ATF5_A*02_, uP_MAPK1_A*03_, and uP_TMEM203_B*07/C*16_, respectively (left panels) compared to a negative HLA-matched control peptide (right panels)

Using isotope-labeled synthetic peptides, we could validate 94% (17/18) of a selected panel of experimental HLA uLigands identifications (Fig. 5a–c, Supplementary Figure 9, Supplementary Table 7), including those derived from the ASNSD1, ATF5, CTNNB1, MAPK1, and TMEM203 uPeptides previously detected by immunoblot experiments (Figs. 2, 4d). To assess the immunogenicity of HLA uLigands, we selected a panel of tumor-associated HLA uLigands presented on the common HLA allotypes HLA-A*02, -A*03, and -B*07. We performed in vitro aAPC-based priming of naïve CD8^+^ T cells from HVs using HLA:peptide monomers of the HLA-A*02-, -A*03-, and -B*07-restricted HLA uLigands P_ATF5_A*02_ (SILQSLVPA), P_MAPK1_A*03_ (ALHQPLVHR), and P_TMEM203_B*07/C*16_ (RSAGPRPAL). De novo priming and expansion of antigen-specific T cells was observed for all three HLA uLigands in 100% of analyzed HVs (*n* = 3) with frequencies of peptide-specific T cells ranging from 0.11–0.83% (mean 0.26%) within the viable CD8^+^ T cell population (Fig. 5d–f). Furthermore, multifunctionality of the induced P_ATF5_A*02_-, P_MAPK1_A*03_-, and P_TMEM203_B*07/C*16_-specific T cells was shown using intracellular cytokine staining (ICS) for IFN-γ and TNF as well as degranulation marker staining for CD107a (Fig. 5g–i) validating these tumor-associated HLA uLigands as uORF-derived T cell epitopes (HLA uEpitopes).

## Discussion

The present work uncovers novel regulatory and immunological functions of uORFs in cancer. We provide evidence for uORF-associated translational regulation of cancer-associated genes as well as uPeptide translation and HLA-restricted presentation as cancer-associated T cell epitopes.

The translation of mRNAs into proteins is a key event in the regulation of gene expression. This is especially true in the cancer setting, as many oncogenes are regulated at this level. Upstream ORFs can impact gene expression of the downstream CDS by triggering mRNA decay or by regulating translation [10, 19–21]. Especially in the context of malignancies defective uORF-mediated regulation may have profound physiological, immunogenic and pathogenic consequences [11, 33, 35]. The mode of action appears to be highly uORF- and uPeptide-specific and may entail co-factor-induced ribosome stalling [37, 65, 66].

Our data revealed uORF-mediated translational regulation in the majority of analyzed cancer-associated transcripts. While previous global analyses demonstrated that uORF carrying transcripts show an overall reduction of translation of the associated downstream main proteins [4, 8, 10, 15, 67–69], data from this and previous projects [15] showed that depending on the individual TLS context, the translation of distinct uAUG and aTIS uORFs may have either activating (*e.g*. ATF5 ΔuAUU.1, JAK2 ΔuCUG.2 and 12, MDM2 ΔuCUG.6) or repressive effects (*e.g.* ASNSD1 ΔuAUG.3, ATF5 ΔuAUG.2, ERBB2 ΔuAUU.2) on CDS translation.

Furthermore, our data provide direct evidence for uPeptide translation both, in vitro and in primary human cells, and demonstrate that the strength of translational regulation and the capability to initiate uPeptide translation can be similar for uAUG and aTIS codons. These data extend and are in line with recent genome-wide ribosome profiling studies confirming the widespread presence and translation of AUG and aTIS uORFs [1, 2, 6, 70, 71]. Dual-luciferase reporter studies of computationally predicted functional uORFs often revealed translational regulatory activity from the respective TLSs [3, 5, 8]. However, some of the high-ranking uAUG or aTIS uORFs analyzed in this study did neither result in changes of luciferase reporter activity nor did they initiate translation of uPeptides under the specific experimental conditions applied here. This highlights the need for individual experimental testing of uORF-mediated translational control and uPeptide functions, as results may vary depending on the cellular context and the global translational and environmental conditions.

In several cases, the actual uPeptide initiating codon was distinct from the one predicted by the highest uORF or uPeptide scores. Furthermore, for some TLSs several alternative uPeptides or incomplete uPeptide ablation after deletion of the initiation codon could be observed. This demonstrates that multiple upstream initiation codons may contribute to uPeptide translation, each conferring individual levels of translational regulation to the transcript. Future studies may systematically search for uPeptide interacting protein-co-factors, metabolites, or small molecule interactors, capable to specifically induce ribosome stalling and to ablate translation of harmful downstream oncogenic proteins.

Genome-scale ribosome profiling studies have allowed for the identification of large populations of uORFs known to undergo translation [1–3, 71]. However, the detectability of the translation products by standard mass spectrometry-based proteomics approaches using tryptic digestion is limited [28, 32, 72–74] due to challenges in detecting trypsin-digested fragments from these short uPeptides, which are presumably characterized by high turnover rates [72]. We here provide direct evidence for the frequent translation and cellular expression of cancer-associated uPeptides by immunoblotting and by immunofluorescence-based microscopy. Some of the ectopically expressed uPeptides, including the MAPK1 CUG.1 and TMEM203 AUG.1 uPeptides, showed highly specific intracellular localizations, suggesting individual functional implications for the respective uPeptides. As the function of an individual uPeptide is not necessarily related to the function of the associated main protein, as exemplified for the ASNSD1 uPeptide [75], it is too early to further speculate on the potential functional implications of the uPeptides detected here. Of note, the rather large EGFP-tag may have influenced both, expression level and localization, but the differences observed across individual uPeptides argue for a predominant impact of the uPeptide causing the specific staining patterns observed. Future work is required to validate and extend on these observations in additional cell types and under various global translational conditions, for example by applying uPeptide-specific antibodies or split-GFP-based techniques [76, 77].

Furthermore, mass spectrometry-based immunopeptidome analysis in primary tumor and healthy tissues identified uORF-derived HLA-presented antigens, validating the observations of uPeptide expression upon ectopic expression in vitro. This demonstrates in accordance with recent immunopeptidomics studies [28, 29, 31], which, however, were mainly limited to cell lines, that uPeptides enter the HLA class I presentation pathway and contribute to the antigen repertoire also in vivo. In contrast to the recently published individualized proteogenomic approaches [29, 31], we here applied an approach using a generic uORF database comprising preselected sequences. Using this strategy, we were able to identify HLA uLigands shared between several samples. We further provide unprecedented evidence for tumor-associated presentation of HLA uLigands in this comprehensive cohort including various different hematological and solid tumor entities as well as different benign tissue samples. The inclusion of benign tissue-derived immunopeptidomes enabled the direct identification of tumor-associated and tumor-enriched HLA uLigands that were never or only rarely presented on benign tissues. This represents a major advantage compared to retrospective approaches using RNA sequencing data [45] facing the drawback that the immunopeptidome is an independent complex layer formed by the antigen presentation machinery and therefore does not necessarily mirror the transcriptome nor the proteome [32]. The direct comparison of benign and malignant tissue-derived immunopeptidome data further is of central importance for the definition of tumor-associated HLA uLigands as it was recently shown that the presentation of HLA uLigands and other cryptic peptides is not restricted to tumor tissues [78]. Tumor-associated cryptic peptides from non-coding regions, including 5′-TLS and 3′-UTR, non-coding RNAs, intronic, intergenic and off-frame regions, represent highly promising targets for anti-tumor immune surveillance as well as the development of immunotherapeutic approaches [30]. In contrast to classical neoepitopes, derived from tumor-specific missense point mutations affecting only one amino acid, these cryptic peptides differ by several amino acids from their respective wild type sequence and thus are even more likely to induce tumor-specific immune responses [79]. Furthermore, their shared presentation across multiple donors and even tumor entities, as so far only described for unmutated tumor-associated self-peptides derived from canonical proteins [44, 47, 80–84], enables a broader applicability compared to private neoantigens. In the future, large cohort studies are needed to analyze HLA uLigand presentation in the evolution of malignant disease (*e.g.* primary diagnosis versus relapse), in different tumor stages, and under anti-cancer treatments.

Focusing on a restricted set of uORFs, this work provides evidence that uORF-derived peptides can be processed into tumor-associated HLA-presented peptides detectable on primary human samples, even without the need for whole-exome and RNA sequencing and the assembly of sample-specific, personalized databases. These data may encourage further studies to screen all of the approximately 190 thousand uAUG and 2.5 million aTIS codons within the human genome [15] and to unravel the whole uORF-derived immunopeptidome landscape in cancer.

At present the pathophysiological role of uORF-derived tumor-associated antigens in cancer immune surveillance is unsettled as spontaneous immune recognition in cancer patients was limited [29]. This might at least in part be due to immunopeptidomics analysis of patient-derived cell lines showing a different pattern of HLA uLigand presentation compared to primary samples. Moreover, high turnover rates of unfunctional uPeptides may limit the uptake by professional antigen-presenting cells, preventing effective priming of naïve T cells. This suggests that further target antigen selection should be based on the knowledge of the functional role of the respective uPeptides. For the understanding of the functional role of uPeptides the investigation of their cellular localization is of particular importance. Chen *et al.* [28] and our data suggest specific and distinct cellular localizations for individual uPeptides highlighting the variety of cellular roles and functions that uORFs might fulfill beyond translational regulation [28, 37, 75, 85].

## Conclusion

The data presented in this work demonstrate the translational regulatory effect of uAUG and aTIS uORFs in cancer-associated transcripts and provide direct evidence for the cellular expression and the HLA-restricted presentation of uORF-derived peptides on primary tissue samples. The data suggest a widespread but largely unexplored regulatory and immunological role of uORFs and uORF-derived peptides in cancer biology. These observations may inspire the development of novel anti-cancer therapies, comprising direct molecular targeting of uORFs or the respective uPeptides as well as immunotherapeutic targeting of tumor-associated HLA uLigands.

### Supplementary Information

Below is the link to the electronic supplementary material.

Supplementary file1 (PDF 1878 KB)

Supplementary file2 (XLSX 29 KB)

## Data Availability

The mass spectrometry data as well as the FASTA have been deposited to the ProteomeXchange Consortium (http://proteomecentral.proteomexchange.org) via the PRIDE partner repository [86] with the dataset identifier PXD025716. Raw files of ovarian carcinoma, benign ovaries [44] and melanoma [45] samples have been downloaded from the PRIDE partner repository (ProteomeXchange Consortium (http://proteomecentral.proteomexchange.org) with the dataset identifier PXD007635 and PXD004894.

## Abbreviations

Aa: Amino acid
aAPC: Artificial antigen-presenting cell
AML: Acute myeloid leukemia
aTIS: Alternative translation initiation site
BMNCs: Bone marrow mononuclear cells
CDS: Protein-coding sequence
CID: Collision-induced dissociation
CLL: Chronic lymphocytic leukemia
CTNNB1: Beta-catenin
FDR: False discovery rate
HLA: Human leukocyte antigen
HLA uEpitope: Upstream ORF-derived T cell epitope
HLA uLigand: Upstream ORF-derived HLA ligand
HPCs: Hematopoietic progenitor cells
HVs: Healthy volunteers
ICS: Intracellular cytokine staining
IDs: Identifications
LC–MS/MS: Liquid chromatography-coupled tandem mass spectrometry
mAb: Monoclonal antibody
MAPK1: Mitogen-activated protein kinase 1
MDM2: Murine double minute 2 homolog
Mel: Melanoma
mORF: Main open reading frame
OvCa: Ovarian carcinoma
OvN: Benign ovaries
PBMCs: Peripheral blood mononuclear cells
RF: Reading frame
RT-qPCR: Real time PCR
SDM: Site-directed mutagenesis
TCRP: Translational control reporter plasmid
TLS: Transcript leader sequence
TMEM203: Transmembrane protein 203
uAUG: Upstream AUG
uORF: Upstream open reading frame
uPeptide: Upstream ORF-encoded peptide
wt: Wild type
ΔuORF: Upstream ORF with mutated start codon
