# Supplementary material for: Upstream open reading frames regulate translation of cancer-associated transcripts and encode HLA-presented immunogenic tumor antigens
Source: Cell Mol Life Sci. 2022 Mar 3;79(3):171. doi: 10.1007/s00018-022-04145-0 (PMC8894207; doi:10.1007/s00018-022-04145-0)
Supplement: Supplementary file 1 — Supplementary file1 (PDF 1878 KB) [file 18_2022_4145_MOESM1_ESM.pdf]

Supplementary Information for

Upstream open reading frames regulate translation of cancer-associated transcripts and encode HLA-presented immunogenic tumor antigens

Annika Nelde[#], Lea Flötotto[#], Lara Jürgens, Laura Szymik, Elvira Hubert, Jens Bauer, Christoph Schliemann, Torsten Kessler, Georg Lenz, Hans-Georg Rammensee, Juliane S. Walz[§], and Klaus Wethmar[§]

[#, §] These authors contributed equally to this work.

Corresponding author: Juliane S. Walz (juliane.walz@med.uni-tuebingen.de) and Klaus Wethmar (klaus.wethmar@ukmuenster.de)

This PDF file includes:

Figures S1 to S9
Tables S1 to S7
Legends for Datasets S1
SI References

Other supplementary materials for this manuscript include the following:

Datasets S1

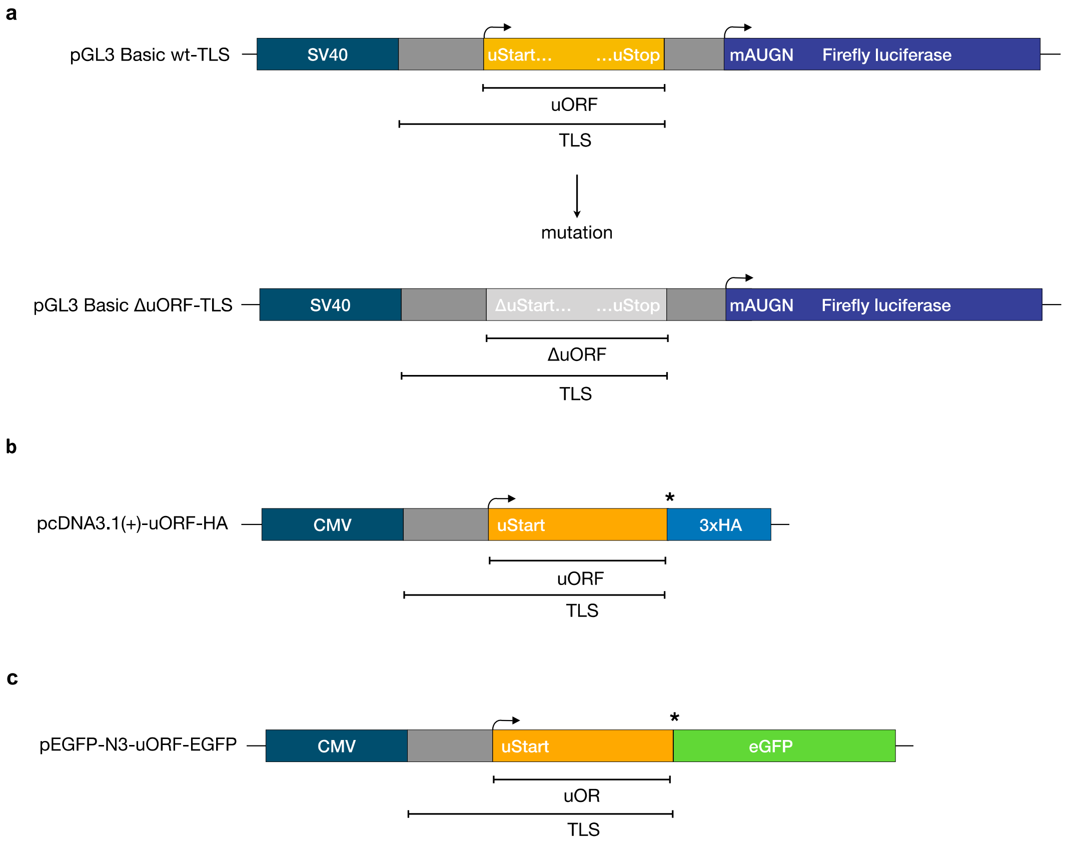

Figure S1: Schematic illustration of the luciferase-based translational control reporter plasmid (TCRP), 3xHA expression vector and eGFP expression vector. a The translational control reporter plasmid[1] enables the insertion of complete TLSs including the endogenous initiation codon of the respective coding sequence (mAUG) plus the surrounding Kozak base +4 (N). Arrows indicate translational initiation sites. The wt TLS was synthesized by GeneArt Gene Synthesis (Life Technologies). The wt uORF start codons were mostly mutated to CUC or occasionally to other non-initiating codons (ΔuORF) by site-directed mutagenesis (for details refer to Supplementary Tab. 1, 6, and 7). **b** The 3x-HA expression vector was used for detection of uPeptides by ligating the uORF under investigation upstream of the 3xHA-tag. *uORF stop codon was deleted to allow continuous translation of the HA-tag. **c** For the detection of uPeptides by immunofluorescence microscopy, TLSs including the uORF were ligated into the pEGFP N3 vector in-frame and upstream of eGFP with a deleted initiation codon. *uORF stop codon was deleted to allow continuous translation of the eGFP-tag.

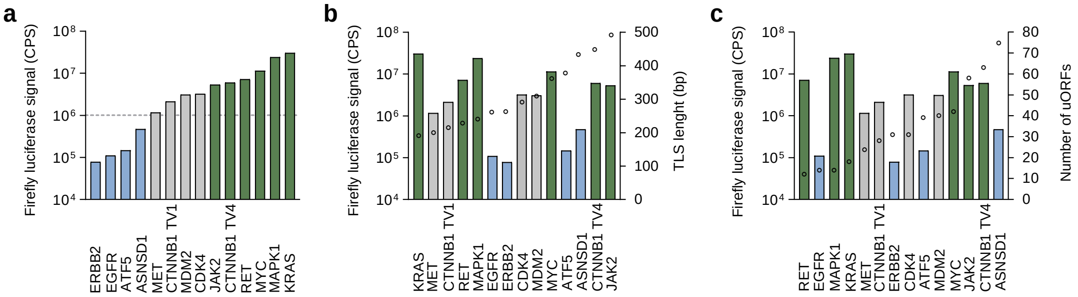

Figure S2: Baseline relative luciferase activity for individual TLS. Bar graphs showing Firefly luciferase signals in counts per second (CPS) of HEK293T cell lysates collected 44 h after transfection with 10 ng of indicated wt TLS containing TCRP. Results are displayed in three different ways, ordered by **a** intensity of the Firefly luciferase signal, **b** the length of the wt TLS (right y-axis), and **c** the number of uAUG plus aTIS codons of indicated TLSs (right y-axis), respectively. Amounts of transfected wt and ∆uORF TCRPs were maintained (grey-shaded bars), increased (blue-shaded bars) or decreased (green-shaded bars) to produce wt TLS luciferase levels of approximately 1,000,000 CPS to enable measurements within the optimal linear range of detection for each TLS construct. Note that for CTNNB1 the uORFs with highest uORF and uPeptid scores mapped to distinct transcript variants. Accordingly, both were selected for experimental analysis.

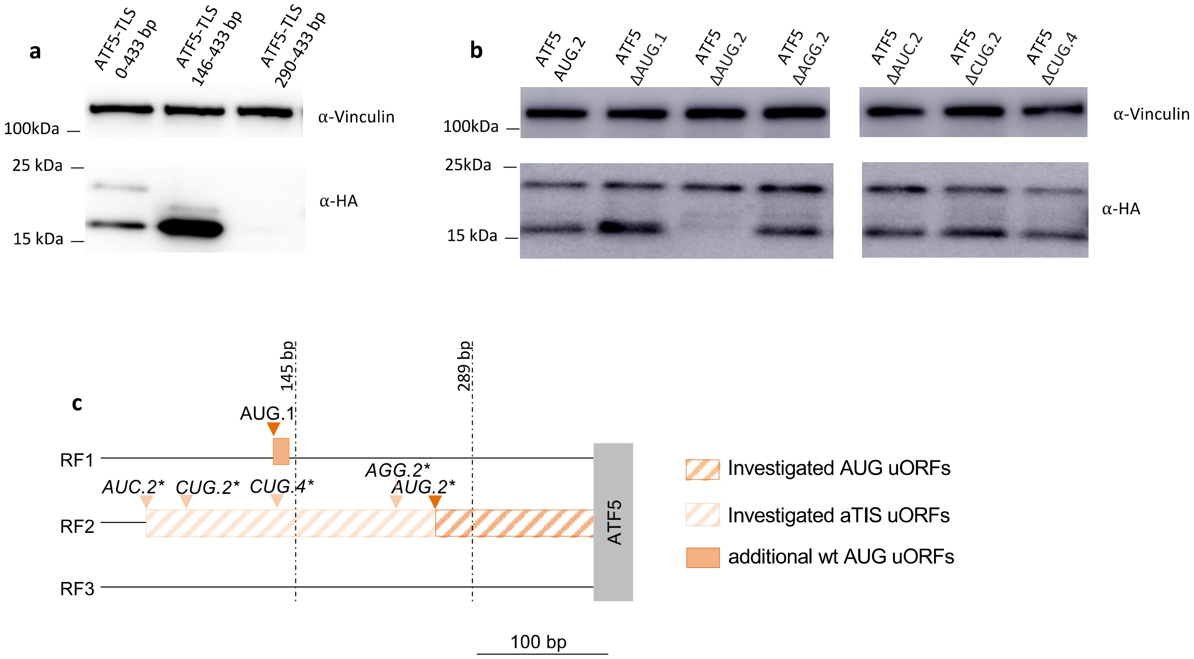

Figure S3: Evidence for multiple uPeptides expressed from ATF5 uAUG.2-tagged TLS. HEK293T cell lysates were prepared 52 h after transfection of expression vectors and after an eight hour MG132 treatment prior to cell lysis. **a** Representative immunoblot showing the expression of 3xHA-tagged full-length TLS (ATF5-TLS 0-433 bp) and two TLS versions shortened from the 5´-end (ATF5-TLS 146-433 bp and ATF5-TLS 290-433 bp). **b** Representative immunoblots showing the expression of multiple ∆uORF TLS variants. **c** Schematic illustration of indicated in-scale ATF5 uAUG.2-tagged TLS displaying multiple analyzed uORFs (striped boxes) in reading frame (RF) 2 and additional wild type AUG uORFs (wt, filled orange boxes) on RF1 to RF3 (black lines).

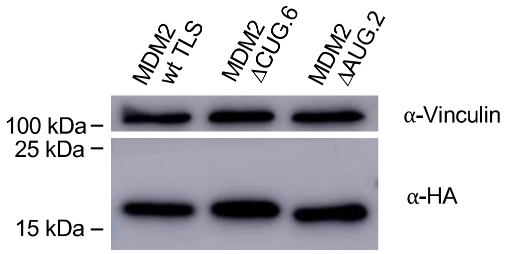

Figure S4: Immunoblot analysis of potential MDM2 uPeptide initiation sites. Representative immunoblot of ≥3 independent experiments using HEK293T cell lysates prepared 52 h after transfection of expression vectors containing indicated triple HA-tagged wt and ΔuORF TLS variants. Eight hours prior to lysis cells were exposed to proteasome inhibitor MG132.

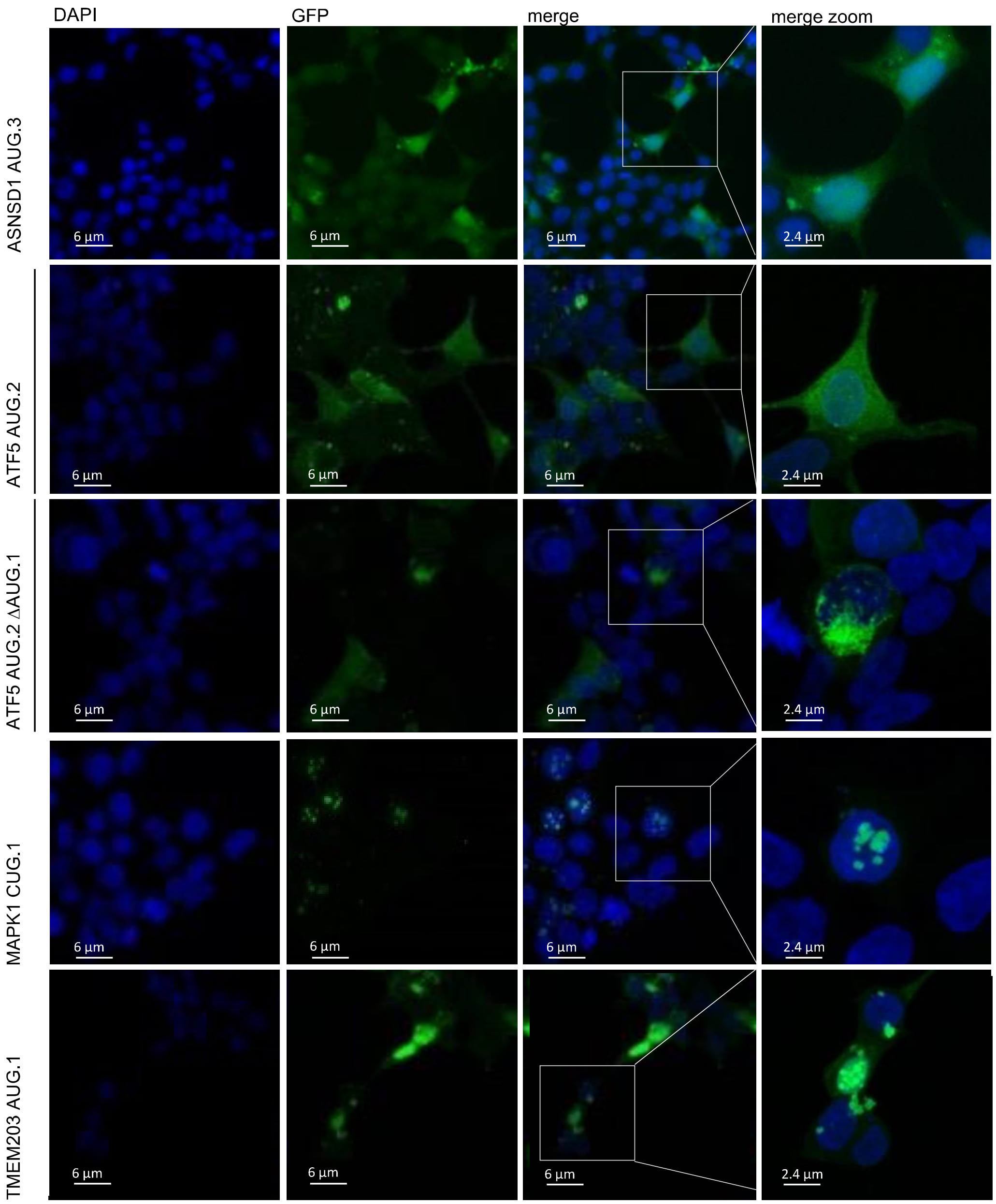

Figure S5: Analysis of EGFP-tagged uPeptide expression and cellular localization. Additional, representative pictures of immunofluorescence based detection of indicated EGFP-tagged uPeptides showing the expression and intracellular localizations 24 h after transfection of HEK293T cells. Upstream peptides were expressed from TLS-EGFP-expression vectors containing the complete 5´-upstream sequence of indicated wt TLSs and an EGFP-tag replacing the uStop codon of the investigated uORF (see Figure S1). The pictures shown here are merged z-stack images.

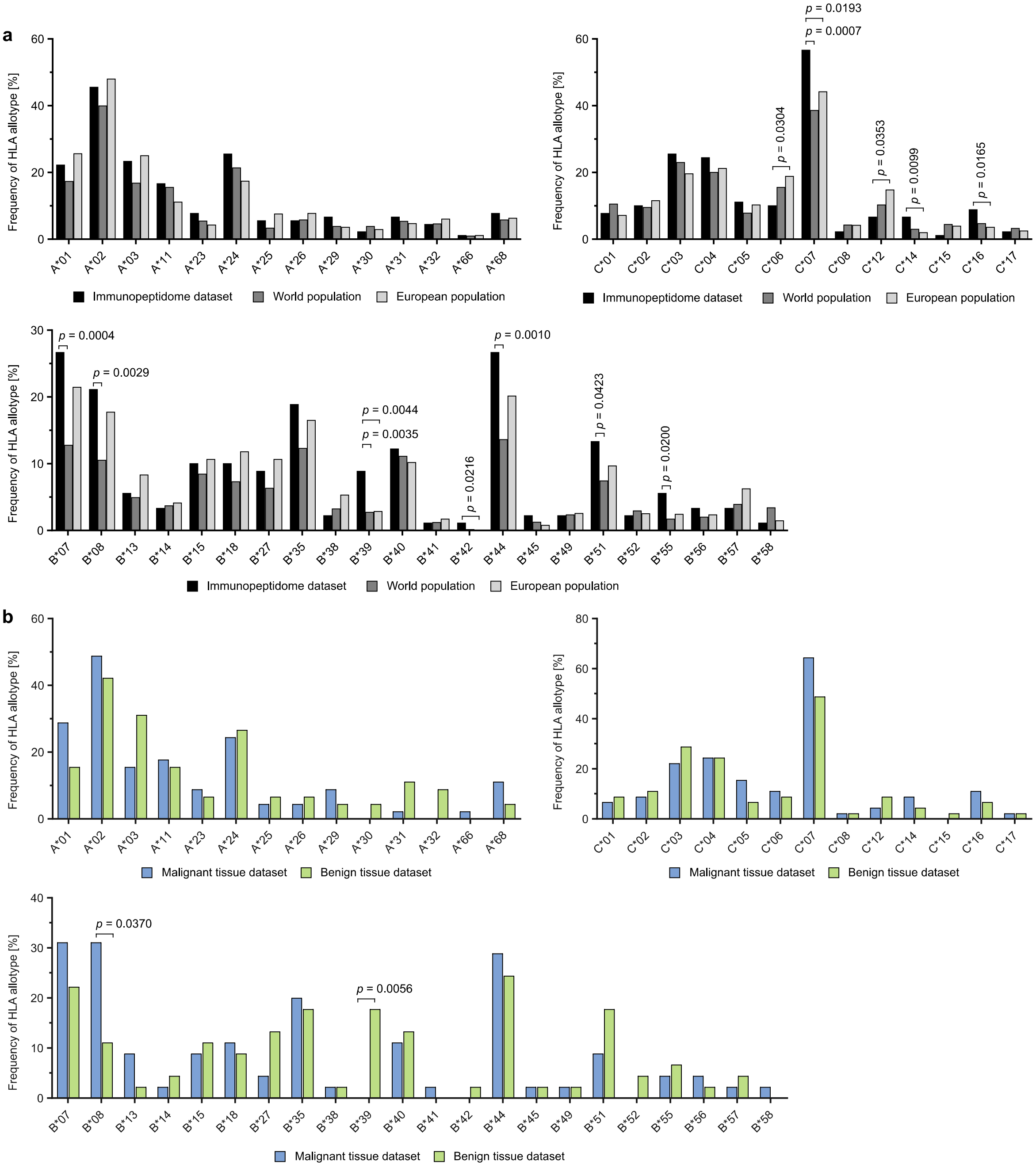

Figure S6: HLA allotype distribution in immunopeptidomics dataset. **a** HLA-A, HLA-B, and HLA-C allotype frequencies in the immunopeptidomics dataset (n = 90) used for mass spectrometry-based analysis of naturally presented, uORF-derived antigens compared to the world and European population. Frequencies of HLA allotypes within the world and European population were calculated according to the IEDB population coverage tool (www.iedb.org) considering all HLA allotypes covered within the immunopeptidomics dataset. P values were determined by Fisher's exact test. **b** HLA-A, HLA-B, and HLA-C allotype frequencies in the malignant (n = 45) and benign (n = 45) tissue datasets. P values were determined by Fisher's exact test.

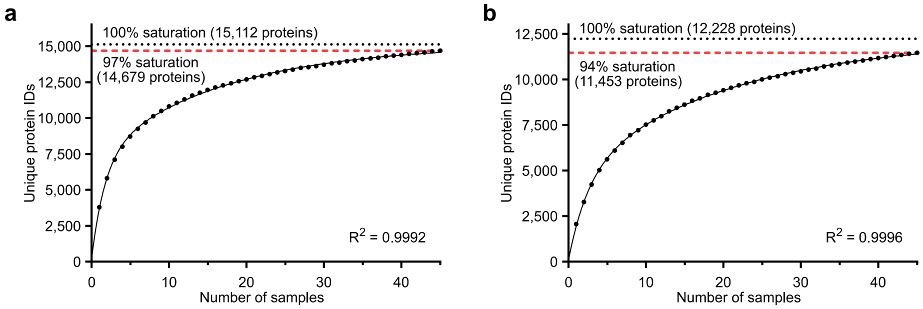

Figure S7: Saturation analysis of HLA ligand source proteins of the malignant and benign tissue datasets. Number of unique HLA ligand source protein identifications shown as function of cumulative immunopeptidome analysis of **a** malignant (n = 45) and **b** benign (n = 45) tissue samples. Exponential regression allowed for the robust calculation (R^2 = 0.9992 for malignant, R^2 = 0.9996 for benign tissue) of the maximum attainable number of different source protein identifications (100% saturation, dotted line). The dashed red line depicts the source proteome coverage achieved in the malignant and benign dataset, respectively. Abbreviation: IDs, identifications.

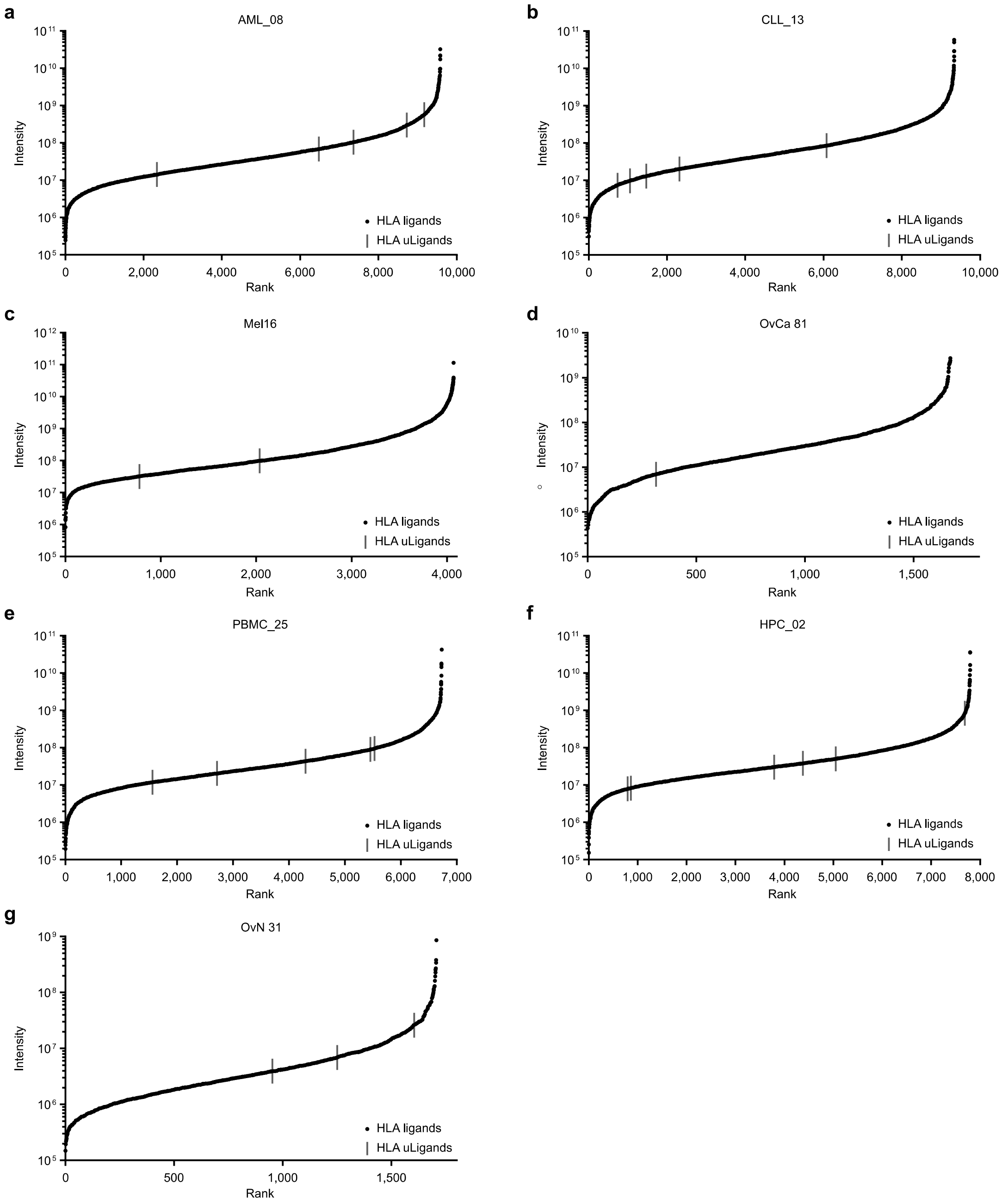

Figure S8: Intensity and distribution of HLA uLigands within the total immunopeptidome. Ranked intensity values of mass spectrometry-acquired data derived from representative examples of **a-d** malignant (**a** AML_08, **b** CLL_13, **c** Mel16, **d** OvCa 81) and **e-g** benign (**e** PBMC_25, **f** HPC_02, **g** OvN 31) samples. Positions of HLA uLigands are projected on the curve.

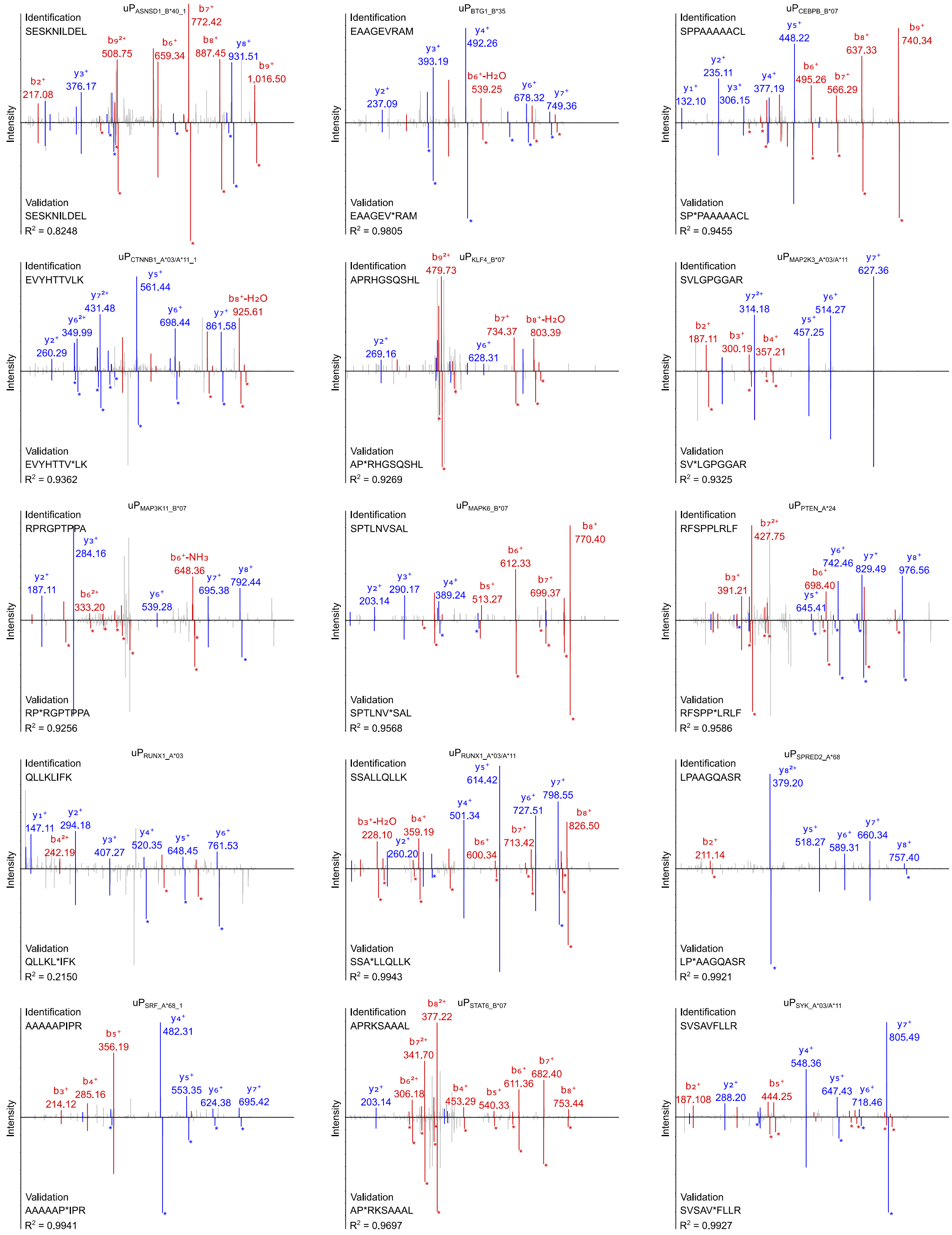

Figure S9: Validation of experimentally eluted peptides by synthetic peptides. Comparison of fragment spectra (m/z on the x-axis) of HLA uLigands eluted from primary samples (identification) to their corresponding synthetic peptides (validation, mirrored on the x-axis) with the calculated spectral correlation coefficient. Identified b- and y-ions are marked in red and blue, respectively. Ions containing isotopic labeled amino acids are marked with asterisks.

Table S1: Sequences of analyzed TLSs.

Name	Sequence
ASNSD1<br>NM_001014431	gctattggtaagactcgcgggaaaagaaagggtgagcgcggctg/ctcgaagcgcgcatgcgctgtggctaatgccgtaggctccttcagggctgagccatcccgcgtgtcttg<br>/ttacgctcggtggaaatg/ctacccagccgagggacgcgaccagaggacagctctgtgctgatccccaccgacaattcgaccccacacaaggaggatctaagcagcaaga<br>ttaaagaacaaaaaattgtggtggatgaactttctaaccttaagaagaataggaaagtatataggcaacaacagaacagcaatatattctttcttgcagaccgaacagaaatgct<br>gtctgagagcaagaatatattggatgaactgaaaaaagaataccaagaaatagaaaacttagacaagaccaaaatcaagaaatagtcaacctgatttcacataacaatgt
ATF5<br>NM_001193646	atccgggagggccgtgctccgccacccagtatatatctgtccccagtccccggggccgcctcatt/cttccctgtcctcggatcacagtctcttctcactacagtgtcgccgcctctgc<br>ctgcgtagccccggccatggctctgtagcctcgacccctttgtgcccccggcccgtctccgcgctcaccacgcctgcgctctccgctcccaccttctttcttcagccgaggccgccg<br>ccgcctctccttgctgcagccatg/ctcgagtcttccactttcgccttg/ctcgtgcctgtcttcgcccacctgagcatcctccagagcctcgtgccagctgctggtgcagcctctcctgtt<br>gccatcagtgcccagcacctgtgctacagccatgtcactcctggcgaccctggggctggagctggacagggccctgctcccagctag
CDK4<br>NM_000075.3	gctagccacctcctgtccgcccctcagcgcatg/ctcggtggcggtcacgtgcccagaacgtccggcgttcgccccgccctcccagtttccgcgcgcctctttg/cttgcagctggt<br>cacatggtg/gcaagggtgggggtgagggggcctctctagcttgcggcctgtgtctatggtcgggccctctgcgtccagctgctccggaccgagctcgggtgtatggggccgtag<br>gaaccggctccggggccccgataacgggccgcccccacagcaccccgggctggcgtgagggtctcccttgatctgagaatggccgcgg
CTNNB1 aTIS<br>NM_001904	aagcctctcggtctgtggcagcagcgttg/gtcgcccggccccgggagcggagagcgaggggaggcggagacggaggaaggtctgaggagcagcttcagtccccgccga<br>gccgccaccgcaggtcgaggacggtcggactcccgcggcgggaggagcctgttcccctgagggtatttgaagtataccatacaactgttttgaaaatccagcgtggacaatgg<br>ctactcaagctgatttga
CTNNB1 uPep<br>NM_001330729	aacgctccgcgctg/ctccgccggtggcggcaggatacagcggcttctgcgcgacttataagagctccttgtgcggcgccattttaagcctctcggtctgtggcagcagcgttggc<br>ccggccccgggagcggagagcgaggggaggcggagacggaggaaggtctgaggagcagcttcagtccccgccgagccgccaccgcaggtcgaggacggtcggactc<br>ccgcggcgggaggagcctgttcccctgagggtatttgaagtataccatacaactgttttgaaaatccagcgtggacaatggctactcaaggctaccttttgctccattttctgctcact<br>cctcctaatggcttggtgaaatagcaaacaagccaccagcaggaatctagtctggatgactgcttctggagcctggatgcagtaccattcttccactgattcactgatttgatgg
EGFR<br>NM_005228	agacgtccgggcagcccccggcgcagcgcggccgcagcagcctccgcccccgcacggtgtgagcgcccgacgcggccgaggcggccggagtcccgagctagccccg<br>gcggccgccgccgcccagaccggacgacaggccacctcgtcggcgtccgcccgagtccccgcctcgccgccaacgccacaaccaccgcgcacggcccccctgactccgtc<br>cagtattg/gtcatcgggagagccggagcgagctcttcggggagcagcgatgcgaccctccgggacggccggggcagcgctcctggcgctgctggctgcgctctgcccggcg<br>agtcgggctctggaggaaaagaaagtttgccaaggcacgagtaa
ERBB2<br>NM_004448	gcgcttg/ttactcccaatcacaggagaaggaggaggtggaggaggagggctgcttgaggaagtataagaatgaagttgtgaagctgagatt/cttcccctccattgggaccgg<br>agaaaccaggggagcccccgggcagccgcgcgccccttcccacggggccctttactgcgccgcgcgcccggcccccacccctcgcagcaccccgcgccccgcgccctcc<br>cagccgggtccagccggagccatg/ctcgggccggagccgcagtgagcaccatggagctggcggccttgtgccgctggggggctcctcctcgccctcttgccccccggagccg<br>cgagcaccacagacaactacctttctacggacgtgggatcctgcaccctcgtctgccccctgcacaaccaagaggtgacagcagaggatggaacacagcggtgtga
JAK2<br>NM_004972	gctagcctgcaggaaggagagaggaagaggagcagaagggggcagcagcggacgccgctaacggcctccctcggcgctg/ctcacaggctgggccggcgcccggctc<br>gcttgggtgttcgcgtcgccacttcggcttctcggccggtcgggcccctcggcccgggcttgcggcgcgcgtcggggctgagggctgctgcggcgcagggagaggcctggtcct<br>cgctgccgagggatgtgagtgggagctgagcccacactggagggcccccgagggcccagcctggaggtcgttcagagccgtgcccgtcccggggcttcgcagaccttgacc<br>cgccgggtaggagccgccc ctg/ctccgggctcgagggcgcgctctggtcgcccgatctgtgtagccggtttcagaagcaggcaacaggaacaagatgtgaactgtttctcttct<br>gcagaaaaagaggctcttcctcctcctcccgcgacggcaaatgttctgaaaaagactctgcatggccgcgg
KRAS<br>NM_004985	tcctaggcggcggccgcggcggcggaggcagcagcggcggcggcagtg/gtcgcggcggcgaaggtggcggcggctcggccagtactcccggcccccgccatttcggac<br>tg/ctcggagcgagcgcggcgcaggcactgaaggcggcggcggggccagaggctcagcggctcccaggtgcgggagagaggcctgctgaaaatgactga
MAPK1<br>NM_138957	gcccctccctccgcccgcccgccggcccgcccgtcagtctggcaggcaggcaggcaatcggtccgagtggctgtcggctcttcagctctcccgctcggcgtcttccttcctcctcc<br>cggtcagcgtcggcggctg/ctccaccggcggcggcgcagtccctgcgggaggggcgacaagagctg/ctcagcggcggccgccgagcgtcgagctcagcgcggcggag<br>gcggcggcggcccggcagccaacatggcggcggcggcggcggcgggcgcgggcccggagatggtccgcgggcaggtgttcgacgtggggccgcgctacaccaacctct<br>cgtacatcggcgagggcgcctacggcatggtgtgctctgcttatga
MDM2<br>NM_002392	gctagcgcaccgcggcgagcttggctgcttctggggcctgtgtggccctgtgtgtcggaaagatg/ctcgagcaagaagccgagcccgaggggcggccgcgacccctctgac<br>cgagatcctg/ctcctgctttcgcagccaggagcaccgtccctccccggattagtgcgtacgagcgcccagtgccctggcccggagagtggaatgatccccgaggcccagggc<br>gtcgtgcttccgcgcgccccgtgaaggaaactggggagtcttgagggacccccgactccaagcgcgaaaaccccggatggagatct
MET<br>NM_000245	gggcgggcggggcgctgggctcagcccggccgcaggtg/gtcacccggaggccctcgccgcccgcggcgccccgagcgctttgtgagcagatg/ctccggagccgagtgg<br>agggcgcgagccagatgcggggcgacagctgacttgctgagaggaggcggggaggcgcggagcgcgcgtgtggtccttgcgccgctgacttctccactggttcctgggcac<br>cgaaagacacttctgagaaattcatcaggctgtgaagcgcgccgtgatgaatatcgaacagagtttaccacagctttgcagcgcgttgacttattcatgg
MYC<br>NM_002467	ggagtttattcataacgcgctctccaagtatacgtggcaatgcgttgctgggttattttaatcattctaggcatcgttttcctccttatgcctctatcattcctccctatctacactaacatccc<br>acgctctgaacgcgcgcccattaatacccttctttcctccactctccctgggactcttgatcaaagcgcggccctttccccagccttagcgaggcgccctgcagcctggtacgcgcg<br>tggcgtggcggtgggcgcgcagtgcgttctcggtgtggagggcagctgttccgcctgcgatgatttatactcacaggacaaggatgcggtttgtcaaacagtactgctacggagg<br>agcagcagagaaagggagagggtttgagagggagcaaaagaaaatggtaggcgcgcgtagttaattcatgcggctctcttactctgtttacatcctagagctagagtgctcgg<br>ctgcccggctgagtctcctccccaccttccccaccctccccaccctccccataagcgcccctcccgggttcccaaagcagagggcgtgggggaaaagaaaaaagatcctctct<br>cgctaatctccgcccaccggccctttataatgcgagggtctggacggctgaggacccccgagctgtgctgctcgcggccgccaccgccgggccccggccgtccctggctcccc<br>tcctgcctcgagaagggcagggcttctcagaggcttggcgggaaaaagaacggagggagggatcgcgctgagtataaaagccggttttcggggctttatctaactcgctgtagt<br>aatt/cttccagcgagaggcagagggagcgagcgggcggccggctagggtggaagagccgggcgagcagagctg/ctccgctgcgggcgtcctgggaagggagatccgg<br>agcgaataggggggcttcgcctctggcccagccctcccgctgatcccccagccagcggtccgcaacccttgccgcatccacgaaactttgcccatagcagcgggcgggcacttt<br>gcactggaacttacaacacccgagcaaggacgcgactctcccgacgcggggaggctattctgcccatttggggacacttccccgccgctgccaggacccgcttctctgaaag<br>gctctccttgcagctgcttagacgatggatttttttcgggtagtggaaaaccagcctcccgcgacgatgcccctcaacgttag
RET<br>NM_020975	gctagcagtcccgcgaccgaagcagggcgcgcagcagcgctgagtgccccggaacgtgcgtcgcgcccccagtg/gtctccgtcgcgtccgccgcgccccgggcggggat<br>ggggcggccagactg/ctcagcgccgcacccgccatccagacccgccggccctagccgcagtccctccagccgtggccccagcgcgcacgggcgatggccgcgg

List of TLS sequences and related transcript identifiers (RefSeqIDs) commercially synthetized (GeneArt' Invitrogen) for dual luciferase reporter assays. Wt and mutated start codons of analyzed uORFs are shown in red and CDS start is underlined.

Table S2: PCR primers for site-directed mutagenesis.

Primer name	Sequence	Primer name	Sequence
ASNSD1 AUG.3 for	gcgctcggtggaaCTAcccagccgaggg	ERBB2 UUG.1 for	gctagcgcgcTTActcccaatcacag
ASNSD1 AUG.3 rev	cgcgagccaccttGATgggtcggctccc	ERBB2 UUG.1 rev	cgatcgcgcgAATgagggttagtgtc
ASNSD1 UUG.2 for	ccatcccgcgtgtCTAgcgctcggtggaaatg	JAK2 CUG.2 for	ctccctcggcgCTCacaggctgggcc
ASNSD1 UUG.2 rev	ggtagggcgcacaGATcgcgagccacctttac	JAK2 CUG.2 rev	gagggagccgcgaGTGtccgacccgg
ASNSD1 CUG.1 for	gtgagcgcggCTCgaagcgcgcatg	JAK2 CUG.12 for	aggagccgcccCTCcgggctcgagg
ASNSD1 CUG.1 rev	cactcgcgccGAGcttcgcgcgtac	JAK2 CUG.12 rev	gccctcgagcccgGAGgggcggctc
ATF5 AUG.2 for	ctccttgctgcagccCTCgagtcttccactttc	KRAS GUG.1 for	ggcggcggcaGTCgcggcggcgaag
ATF5 AUG.2 rev	gaggaacgacgtcggGAGctcagaaggtgaaag	KRAS GUG.1 rev	ccgccgccgtCAGcgccgccgcttc
ATF5 UUG.3 for	cttccactttcgccCTCgtgcctgtcttcgcc	KRAS CUG.1 for	ccatttcggaCTCggagcgagcgcg
ATF5 UUG.3 rev	gaaggtgaaagcggGAGcacggacagaagcgg	KRAS CUG.1 rev	ggtaaagcctGAGcctcgctcgcgc
ATF5 AUU.1 for	cggggccgcctcCTTccctgtcctc	MAPK1 CUG.3 for	gtcagcgtcggcggCTCcaccggcggc
ATF5 AUU.1 rev	gccccggcGGAggaagggacaggag	MAPK1 CUG.3 rev	cagtcgcagccgccCACgtggccgccg
ATF5 AGG.2 for	ctttcttcagccgCGGccgccgccgcctc	MAPK1 CUG.5 for	gcgacaagagCTCagcggcggccgc
ATF5 AGG.2 rev	gaaagaagtcggcGCCggcggcggcggag	MAPK1 CUG.5 rev	cgctgttctcGAGtcgccgccggcg
ATF5 ACG.1 for	tccgcgctcaccCCGcctgcgctctc	MAPK1 CUG.4 for	gcgcagtccCTCcgggaggggcg
ATF5 ACG.1 rev	aggcgcgagtggGGCggacgcgagag	MAPK1 CUG.4 rev	cgcgtcaggGAGgccctccccgc
ATF5 AGG.1 for	atccgggAGCgccgtgctccgccac	MAPK1 AGG.5 for	ctcagcgcggcggTGGcggcggcggccc
ATF5 AGG.1 rev	taggcccTCGcggcacgaggcggtg	MAPK1 AGG.5 rev	gagtcgcgccgccACCgccgccgccggg
ATF5 GUG.1 for	cgggagggccGAGctccgccaccc	MAPK1 CUG.1 for	cgcccgtcagtCCGgcaggcaggcag
ATF5 GUG.1 rev	gccctcccggCTCgaggcggtggg	MAPK1 CUG.1 rev	cgggcagtcaGGCcgtccgtccgtc
ATF5 AUC.2 for	cacccagtatatAACtgtccccagtcc	MAPK1 AGG.2 for	gtctggcaggcAGAcaggcaatcgg
ATF5 AUC.2 rev	gtgggtcatataTTGacaggggtcagg	MAPK1 AGG.2 rev	cagaccgtccgTCTgtccgttagcc
ATF5 CUG.2 for	cgcctcattccCTCtcctcggatcacag	MAPK1 AUC.1 for	gcaggcaggcaCTCggtccgagtgg
ATF5 CUG.2 rev	gcggagtaaggGAGaggagcctagtgtc	MAPK1 AUC.1 rev	cgtccgtccgtGAGccaggctcacc
ATF5 CUG.4 for	ccgcctctgcCTCcgtagccccggc	MDM2 AUG.1 for	ctgtgtgtcggaaagCTCgagcaagaagccga
ATF5 CUG.4 rev	ggcggagacgGAGgcatcggggcg	MDM2 AUG.1 rev	gacacacagcctttcGAGctcgttcttcggctc
CDK4 AUG.1 for	cccctcagcgcCTCggtggcggtcac	MDM2 CUG.6 for	gaccgagatcCTCctgctttcgcag
CDK4 AUG.1 rev	ggggagtcgcgGAGccaccgccagtg	MDM2 CUG.6 rev	ctggctctagGAGgacgaaagcgtc
CDK4 GUG.3 for	gctggtcacatgGCAagggtgggggtg	MDM2 CUG.7 for	ccgagatcctgCTTctttcgcagccagg
CDK4 GUG.3 rev	cgaccagtgtaccGTTcccaccccac	MDM2 CUG.7 rev	ggctctaggacGAAgaaagcgtcggtcc
CDK4 UUG.1 for	gtttccgcgcgcctctCTTgcagctggtcacatg	MDM2 GUG.5 for	ctccccggattaGTTcgtacgagcgccc
CDK4 UUG.1 rev	caaaggcgcgcggagaGAAcgtcgaccagtgtac	MDM2 GUG.5 rev	gaggggcctaatCAAgcatgctcgcggg
CTNNB1 UUG.1 for	gtggcagcagcgGTCgcccggccccg	MDM2 AUG.2 for	ggagagtggaCTCatccccgaggc
CTNNB1 UUG.1 rev	caccgtcgtcgcCAGcgggccggggc	MDM2 AUG.2 rev	cctctcacctGAGtaggggctccg
CTNNB1 CUG.4 for	gagcctgttcccCAGagggtatttgaag	MDM2 AUC.2 for	gagagtggaatgACCcccgaggcccagg
CTNNB1 CUG.4 rev	ctcggacaagggGTCtcccataaacttc	MDM2 AUC.2 rev	ctctcaccttacTGGgggctccgggtcc
CTNNB1 UUG.3 for	catacaactgttTCGaaaatccagcg	MET AUG.1 for	gctttgtgagcagCTCcggagccgagtgg
CTNNB1 UUG.3 rev	gtatgttgacaaAGCttttaggtcgc	MET AUG.1 rev	cgaaacactcgtcGAGgcctcggctcacc
CTNNB1 CUG.1 for	aacgctccgcgCTCcgccggtggcggcagg	MET GUG.1 for	gctagcgcagGTCacccggaggccc
CTNNB1 CUG.1 rev	ttgcgaggcgcCACgcggccaccgccgtcc	MET GUG.1 rev	cgatcgcgtcCAGtgggcctccggg
EGFR UUG.1 for	gactccgtccagtaGTCatcgggagagccgg	MYC CUG.2 for	ctcgctgtagtaCTTccagcgagagg
EGFR UUG.1 rev	ctgaggcaggtcatCAGtagccctctcggcc	MYC CUG.2 rev	gagcgacatcatGAAggtcgctctcc
ERBB2 AUG.2 for	cagccggagccCTCgggccggagccgcagtgag	MYC AUU.1 for	gcgagcagagCTCcgctgcgggcgtc
ERBB2 AUG.2 rev	gtcggcctcggGAGcccggcctcggcgtcactc	MYC AUU.1 rev	cgctcgtctcgagGCGacgcccgcag
ERBB2 AUU.1 for	gttgtgaagctgagCTTcccctccattg	RET CUG.2 for	gcggccagaCTAagcgccgcacccg
ERBB2 AUU.1 rev	caacacttcgactcGAAggggaggtaac	RET CUG.2 rev	cccgccggtctGATtcgcggcgtgg
TMEM203 AUG.1 for	gtgggggcACCgcgtccgatcgagg	RET GUG.2 for	tcgcgcccccaGTCtccgtcgcgtc
TMEM203 AUG.1 rev	cacccccgTGGcgcaggctagctcc	RET GUG.2 rev	cgcgggggtCAGaggcagcgcaggc

List of oligonucleotides used to mutate indicated uORF start codons by site-directed mutagenesis. Mutated codons are shown in capital letters and the modified bases are marked in red.

Table S3: Characteristics of selected uORF initiation codons for functional testing.

Gene / RefSeq transcript	Initiation codon (wt>ΔuORF)	Stop codon +/- CDS position	uORF score	uPeptide score	Kozak consensus
ASNSD1 NM_001014431	AUG.3>CUA	UAG.6 -21 bp	n.a.	n.a.	adequate
	UUG.2>UUA	UAG.6 -21 bp	14.8	0.00013	adequate
	CUG.1>CUC	UAA.2 -366 bp	14.7	0.00198	adequate
ATF5 NM_001193646	AUG.2>CUC	UAG.3 +56 bp	n.a.	n.a.	strong
	UUG.3>CUC	UAG.3 +56 bp	21.3	0.00031	strong
	AUU.1>CUU	UAG.2 -234 bp	18.8	0.59359	weak
CDK4 NM_000075.3	AUG.1>CUC	UGA.4 -7 bp	23.9	0.00088	adequate
	GUG.3>GCA	TAG.1 -153 bp	25.0	0.00029	adequate
	UUG.1>CUU	TAG.1 -153 bp	24.8	0.00198	adequate
CTNNB1 NM_001904 (TV1) NM_001330729 (TV4)	UUG.1>GUC	UGA.5 +22 bp	23.7	0.00013	strong
	CUG.1>CUC	UAA.2 -365 bp	16.4	0.59359	adequate
EGFR NM_005228	UUG.1>GUC	UAA.1 +107 bp	24.1	0.00088	adequate
ERBB2 NM_004448	AUG.2>CUC	UGA.4 -5 bp	n.a.	n.a.	strong
	AUU.2>CUU	UGA.7 +247 bp	13.5	0.00029	adequate
	UUG.1>UUA	UAA.1 -199 bp	9.2	0.00031	weak
JAK2 NM_004972	CUG.2>CUC	UGA.2 -316 bp	20.4	0.00088	adequate
	CUG.12>CUC	UAG.2 -111 bp	16.7	0.90260	weak
KRAS NM_004985	GUG.1>GUC	UGA.1 -60 bp	25.5	0.16448	strong
	CUG.1>CUC	UGA.4 +8 bp	24.3	0.62603	strong
MAPK1 NM_138957	CUG.3>CUC	UGA.1 -60 bp	25.4	0.00081	weak
	CUG.5>CUC	UGA.2 +131 bp	25.1	0.00088	adequate
MDM2 NM_002392	AUG.1>CUC	UGA.1 -186 bp	19.8	0.00198	strong
	CUG.6>CUC	UGA.3 -52 bp	20.0	0.00088	adequate
MET NM_000245	AUG.1>CUC	UGA.3 -99 bp	0.5	0.00029	weak
	GUG.1>GUC	UGA.3 -99 bp	15.8	0.00198	weak
MYC NM_002467	CUG.2>CUC	UAG.7 +62 bp	21.4	0.00013	adequate
	AUU.1>CUU	UAG.2 -307 bp	18.6	0.32523	adequate
RET NM_020975	CUG.2>CUC	UAG.1 -39 bp	7.5	0.76758	adequate
	GUG.3>GUC	UGA.2 -74 bp	7.3	0.90261	weak

Characteristics of selected uORF initiation codons for functional testing. Note that several of the selected genes did not contain uAUG codons in any RefSeq transcript variant and that in some cases highest uORF and uPeptide scores mapped to the same codon. The uAUGs of ASNSD1, ATF5 and ERBB2 were selected based on previous experimental data as no uORF score was available in McGillivray *et al.*[2]. Of all uORFs ranked according in the paper of McGillivray *et al.* the top 50% of the uORFs have a uORF score ≥ 10.5, the top 10% have a uORF score ≥ 21.0 and the top 1% have a uORF score ≥ 25.3. For the uPeptide score the top 50% have a uPeptide score ≥ 0.0003, the top 10% have a uPeptide score ≥ 0.002 and the top 1% have a uPeptide score ≥ 0.90. The quality of the Kozak consensus sequence was categorized as optimal (GCCRCCAUGG), strong (NNNRNNAUGG), adequate (NNNRNNAUG[A/C/U] and NNN[C/U]NNAUGG), and weak (NNN[C/U]NNAUG[A/C/U]) with N representing any base and R representing a purine base[3].

Table S4: Sample characteristics of malignant tissue samples.

USN	HLA typing	Mass spectrometer	HLA ligand IDs	HLA uLigand IDs
AML_01	A*03:01, A*26:01, B*07:02, B*35:01, C*04:01, C*07:02	Lumos	5,643	7
AML_02	A*02:01, A*23:01, B*44:02, B*44:03, C*02:02, C*04:01	Lumos	5,944	2
AML_03	A*02:01, A*11:01, B*08:01, B*57:01, C*07:01	Lumos	7,912	3
AML_04	A*02:01, A*11:01, B*35:01, B*44:02, C*04:01, C*05:01	Lumos	8,827	5
AML_05	A*01:01, A*29:02, B*08:01, B*44:03, C*07:01, C*16:01	Lumos	6,035	0
AML_06	A*03:01, A*23:01, B*18:01, B*44:02, C*05:01, C*12:03	Lumos	7,056	4
AML_07	A*01:01, B*07:02, B*08:01, C*07:01, C*07:02	Lumos	6,919	9
AML_08	A*02:01, A*02:05, B*51:01, B*58:01, C*14:02, C*03:02	Lumos	9,779	5
AML_09	A*11:01, B*07:02, B*44:03, C*07:02, C*16:01	Lumos	11,248	14
AML_10	A*11:01, A*68:01, B*15:01, B*51:01, C*03:03, C*14:02	Lumos	8,950	9
AML_11	A*01:01, B*08:01, B*13:02, C*06:02, C*07:01	Lumos	4,976	2
AML_12	A*03:01, A*66:01, B*14:01, B*41:02, C*08:02, C*17:01	Lumos	5,160	6
AML_13	A*02:05, A*24:02, B*07:02, B*55:01, C*01:02, C*07:02	Lumos	4,667	4
AML_14	A*02:01, A*31:01, B*07:02, C*07:02	Lumos	4,916	7
AML_15	A*02:01, A*24:02, B*07:02, B*51:01, C*07:02, C*14:02	Lumos	5,004	5
CLL_01	A*02:01, B*15:01, B*56:01, C*03:04, C*01:02	Lumos	7,945	3
CLL_02	A*02:01, B*07:02, B*18:01, C*03:04, C*06:02	Lumos	6,029	3
CLL_03	A*02:01, A*11:01, B*35:01, B*40:01, C*04:01, C*03:04	Lumos	8,640	8
CLL_04	A*02:01, A*68:01, B*38:01, B*51:01, C*12:03, C*14:02	Lumos	6,742	6
CLL_05	A*03:01, A*24:02, B*35:01, C*04:01	Lumos	4,386	2
CLL_06	A*02:01, A*24:02, B*07:02, B*44:02, C*07:02, C*05:01	Lumos	8,816	7
CLL_07	A*02:01, A*11:01, B*07:02, B*15:01, C*07:02, C*03:04	Lumos	7,529	6
CLL_08	A*02:01, A*24:02, B*07:02, B*13:02, C*06:02, C*07:02	Lumos	4,538	1
CLL_09	A*02:01, A*24:02, B*13:02, B*44:02, C*05:01, C*06:02	Lumos	7,657	4
CLL_10	A*01:01, A*24:02, B*15:01, B*40:01, C*02:02, C*07:02	Lumos	5,303	3
CLL_11	A*01:01, A*02:01, B*08:01, B*27:02, C*02:02, C*07:01	Lumos	8,521	3
CLL_12	A*01:01, A*24:02, B*07:02, B*08:01, C*07:02	Lumos	4,924	1
CLL_13	A*02:01, A*29:02, B*44:02, C*05:01, C*16:01	Lumos	9,540	5
CLL_14	A*01:01, A*24:02, B*08:01, B*35:01, C*04:01, C*07:02	Lumos	3,621	1
CLL_15	A*11:01, A*68:01, B*08:01, B*35:01, C*04:01, C*07:02	Lumos	4,393	3
OvCa 104	A*03:01, B*07:02, B*35:08, C*04:01, C*07:02	XL	2,825	5
OvCa 105	A*26:01, A*68:01, B*18:01, B*55:01, C*03:03, C*07:01	XL	2,498	8
OvCa 109	A*02:01, A*23:01, B*40:01, B*49:01, C*07:01, C*03:04	XL	2,836	1
OvCa 111	A*01:01, A*25:01, B*08:01, B*44:02, C*05:01, C*07:01	XL	3,996	1
OvCa 114	A*29:02, B*44:03, C*16:01	XL	3,731	2
OvCa 13	A*02:01, B*35:01, B*40:01, C*03:04, C*04:01	XL	1,756	0
OvCa 45	A*01:01, A*23:01, B*08:01, B*44:02, C*04:01, C*07:02	XL	2,239	0
OvCa 66	A*11:01, A*29:02, B*18:01, B*44:03, C*05:01, C*16:01	XL	2,586	2
OvCa 81	A*02:01, B*45:01, B*56:01, C*07:02, C*01:02	XL	1,684	1
OvCa 99	A*02:01, A*24:02, B*13:02, B*40:01, C*03:04, C*06:02	XL	3,794	1
Mel12	A*01:01, B*08:01, C*07:01	qExactive	5,026	1
Mel15	A*03:01, A*68:01, B*27:05, B*35:03, C*02:02, C*04:01	qExactive	25,249	54
Mel16	A*01:01, A*24:02, B*07:02, B*08:01, C*07:01, C*07:02	qExactive	4,205	2
Mel5	A*01:01, A*25:01, B*08:01, B*18:01	qExactive	5,584	0
Mel8	A*01:01, A*03:01, B*07:02, B*08:01, C*07:01, C*07:02	qExactive	8,303	8

Sample characteristics and peptide yields of malignant samples included in immunopeptidome analysis. Abbreviations: USN, uniform sample number; IDs, identifications; AML, acute myeloid leukemia; CLL, chronic lymphocytic leukemia; OvCa, ovarian carcinoma; Mel, melanoma.

Table S5: Sample characteristics of benign tissue samples.

USN	HLA typing	Mass spectrometer	HLA ligand IDs	HLA uLigand IDs
PBMC_01	A*03:01, A*24:02, B*07:02, B*51:01, C*01:02, C*07:02	XL	1,482	1
PBMC_02	A*02:01, A*03:01, B*35:01, B*39:01, C*04:01, C*07:02	XL	1,206	1
PBMC_03	A*03:01, A*29:02, B*44:02, B*51:01, C*01:02, C*16:01	XL	1,400	1
PBMC_04	A*02:01, A*03:01, B*07:02, B*39:01, C*07:02	XL	1,162	0
PBMC_05	A*11:01, A*31:01, B*27:05, B*52:01, C*02:02	XL	1,139	1
PBMC_06	A*01:01, A*03:01, B*07:02, B*51:01, C*07:02, C*14:02	XL	1,319	1
PBMC_07	A*02:01, A*24:02, B*35:01, B*39:01, C*02:02, C*04:01	XL	1,429	1
PBMC_08	A*02:01, A*11:01, B*27:05, B*39:01, C*07:02	XL	1,261	0
PBMC_09	A*03:01, B*51:01	XL	684	1
PBMC_10	A*02:01, A*31:01, B*27:05, B*40:01, C*03:04	XL	1,156	4
PBMC_11	A*03:01, A*68:01, B*49:01, B*51:01, C*01:02	XL	1,178	3
PBMC_12	A*02:01, A*03:01, B*44:02, B*51:01, C*15:02	XL	1,673	0
PBMC_13	A*02:01, A*11:01, B*40:01, B*44:02, C*03:04	XL	1,196	0
PBMC_14	A*23:01, A*68:01, B*35:01, B*39:01, C*04:01, C*12:03	XL	1,162	3
PBMC_15	A*25:01, A*30:01, B*13:02, B*51:01, C*06:02, C*07:02	XL	1,468	2
PBMC_16	A*25:01, A*29:02, B*08:01, B*45:01, C*06:02, C*07:02	XL	1,314	1
PBMC_17	A*11:01, A*30:01, B*08:01, B*39:01, C*07:02	XL	1,440	1
PBMC_18	A*01:01, A*26:01, B*15:01, B*40:01, C*03:04	XL	1,027	0
PBMC_19	A*02:01, A*11:01, B*44:02, B*55:01, C*03:04, C*05:01	XL	1,871	1
PBMC_20	A*02:01, A*24:02, B*15:01, B*57:01, C*03:04, C*06:02	XL	1,153	1
PBMC_21	A*02:01, A*24:02, B*35:01, B*55:01, C*03:04, C*04:01	Lumos	4,541	1
PBMC_22	A*01:01, A*02:01, B*07:02, C*07:02	Lumos	3,081	1
PBMC_23	A*11:01, A*32:01, B*18:01, B*44:02, C*05:01, C*07:02	Lumos	4,174	1
PBMC_24	A*01:01, A*23:01, B*44:02, C*04:01	Lumos	2,541	0
PBMC_25	A*03:01, B*07:02, B*35:01, C*04:01, C*07:02	Lumos	6,883	5
PBMC_26	A*01:01, A*03:01, B*08:01, B*44:02, C*07:02, C*16:01	Lumos	4,727	3
PBMC_27	A*02:01, A*31:01, B*18:01, B*40:01, C*03:04, C*07:02	Lumos	3,518	2
PBMC_28	A*11:01, B*44:02, B*55:01, C*03:04, C*05:01	Lumos	5,771	11
PBMC_29	A*24:02, A*32:01, B*07:02, B*14:02, C*07:02	Lumos	5,969	5
PBMC_30	A*02:01, B*40:01, B*56:01, C*01:02, C*03:04	Lumos	5,740	7
HPC_01	A*03:01, B*07:02, C*07:02	Lumos	3,664	6
HPC_02	A*02:01, A*24:02, B*27:05, B*44:02, C*02:02, C*04:01	Lumos	7,990	6
HPC_03	A*02:01, B*35:01, B*39:01, C*04:01	Lumos	4,954	1
HPC_04	A*01:01, A*32:01, B*08:01, B*57:01, C*06:02, C*07:02	Lumos	2,518	0
HPC_05	A*24:02, A*31:01, B*07:02, B*44:02, C*04:01, C*07:02	Lumos	3,672	2
OvN 28	A*02:01, B*35:01, B*40:01, C*03:04, C*04:01	XL	1,350	0
OvN 31	A*03:01, A*31:01, B*18:01, B*27:05, C*02:02, C*07:01	XL	1,722	3
OvN 38	A*24:02, A*32:01, B*39:01, B*44:02, C*07:02, C*16:01	XL	1,975	0
OvN 43	A*23:01, A*24:02, B*27:05, B*42:02, C*02:02, C*17:01	XL	1,678	2
OvN 44	A*02:01, A*24:02, B*07:02, B*51:01, C*07:02, C*14:02	XL	1,532	0
OvN 50	A*02:01, A*26:01, B*15:01, B*52:01, C*03:04, C*12:03	XL	1,484	0
OvN 51	A*02:01, A*24:02, B*07:02, B*14:02, C*07:02, C*08:02	XL	1,637	1
OvN 52	A*03:01, A*25:01, B*15:01, B*18:01, C*03:03, C*12:03	XL	2,955	2
OvN 56	A*03:01, A*26:01, B*35:01, B*38:01, C*04:01, C*12:03	XL	2,122	0
OvN 57	A*01:01, A*24:02, B*08:01, B*15:01, C*03:04, C*07:02	XL	3,235	1

Sample characteristics and peptide yields of benign tissue samples included in immunopeptidome analysis. Abbreviations: USN, uniform sample number; IDs, identifications; PBMCs, peripheral blood mononuclear cells; HPC, hematopoietic progenitor cells; OvN, benign fallopian tube samples.

Table S6: Details on experimentally detected uPeptides.

Gene	Expected start codon	Analyzed start codon mutants	Amino acid sequence	Theoretical size [kDa]	Theoretical size including 3x HA-tag [kDa]	Approx. size detected [kDa]
ASNSD1 NM_001014431	**AUG.3**	AUG.3	**M**PSRGTRPEDSSVLIPTDNSTP HKEDLSSKIKEQKIVVDELSNLK KNRKVYRQQQNSNIFFLADRTE MLSESKNILDELKKEYQEIENLD KTKIKK-	23.3	31.8	**17**
ATF5 NM_001193646	**AUG.2**	AUG.2, AUC.2, ACG.1, AGG.1, AGG.2, CUG.2, CUG.4, , GUG.1 ,	**M**ESSTFALVPVFAHLSILQSLVP AAGAASPVAISAQHLCYSHVTP GDPGAGAGQGPAPS-	15.6	24.0	**18**
CTNNB1 NM_001904	UUG.1	UUG.1, **UUG.3**, CUG.4	LARPRERRARGGGDGGRSEE QLQSPPSRHRRSRTVGLPRRE EPVPLRVFEVYHTTV**L**KIQRGQ WLLKLI-	16.8	25.2	**17**
MAPK1 NM_138957	CUG.5	**CUG.1,** CUG.4, CUG.5, AGG.2, AGG.5, AUC.1	***L****AGRQAIGPSGCRLFSSPARRL PSSSRSASAAAPAAAQSLREG RQE*LSGGRRASSSARRRRRP GSQHGGGGGGGRGPGDGPR AGVRRGAALHQPLVHRRGRLR HGVLCL-	15.5	24.0	**20**
MDM2 NM_002392	CUG.6	**AUC.2,** AUG.2, GUG.5, CUG.6, CUG.7	LLLSQPGAPSLPGLVRTSAQCP GPESGM**I**PEAQGVVLPRAP-	10.6	19.1	**17**

Details on experimentally detected uPeptides. Initiation codons and amino acids representing the experimentally identified uPeptide initiation sites are underlined and shown in bold letters. For MAPK1 the peptide sequence upstream of the predicted initiation codon is shown in italics. Sequences of detected HLA uLigands are shown in blue letters.

Table S7: HLA uLigands selected for validation

Peptide ID	Sequence	HLA restriction	Source uORF	HLA uLigand group	Freq. immunopeptidome dataset (n = 90)	Freq. malignant dataset (n = 45)	Freq. benign dataset (n = 45)
$uP_{TMEM203\_B*07/C*16}$	RSAGPRPAL	B*07/C*16	TMEM203 NM_053045.2 CUG.1, CUG.2, UUG.2	tumor-associated	8.9%	17.8%	0.0%
uP_{ATF5_A*02}	SILQSLVPA	A*02	ATF5 NM_001193646 AUG.2, UUG.3	tumor-associated	4.4%	8.9%	0.0%
$uP_{SRF\_A*68\_1}$	AAAAAPIPR	A*68	SRF NM_003131.4 AUG.1	tumor-associated	4.4%	8.9%	0.0%
$uP_{MAP3K11\_B*07}$	RPRGPTPPA	B*07	MAP3K11 NM_002419 AUG.1	tumor-associated	3.3%	6.7%	0.0%
uP_{SPRED2_A*68}	LPAAGQASR	A*68	SPRED2 NM_181784.3 CUG.14, AUG.4	tumor-associated	3.3%	6.7%	0.0%
uP_{KLF4_B*07}	APRHGSQSHL	B*07	KLF4 NM_001314052 AUG.1	tumor-associated	2.2%	4.4%	0.0%
$uP_{MAP2K3\_A*03/A*11}$	SVLGPGGAR	A*03/A*11	MAP2K3 NM_001316332.2 AUG.1	tumor-associated	2.2%	4.4%	0.0%
uP_{MAPK1_A*03}	ALHQPLVHR	A*03	MAPK1 NM_138957 CUG.5	tumor-associated	2.2%	4.4%	0.0%
uP_{RUNX1_A*03}	QLLKLIFK	A*03	RUNX1 NM_001001890 AUG.6	tumor-associated	2.2%	4.4%	0.0%
uP_{STAT6_B*07}	APRKSAAAL	B*07	STAT6 NM_003153 AUG.2	tumor-enriched	11.1%	17.8%	4.4%
$uP_{SYK\_A*03/A*11}$	SVSAVFLLR	A*03/A*11	SYK NM_001174167.3 AUG.1	tumor-enriched	7.8%	11.1%	4.4%
uP_{CEBPB_B*07}	SPPAAAAACL	B*07	CEBPB NM_001285878 AUG.2, AUA.1	tumor-enriched	6.7%	8.9%	4.4%
uP_{BTG1_B*35}	EAAGEVRAM	B*35	BTG1 NM_001731.2 CUG.2, CUG.4, TTG.2, ACG.1	high frequent	11.1%	13.3%	8.9%
uP_{PTEN_A*24}	RFSPPLRLF	A*24	PTEN NM_000314 AUG.3	high frequent	11.1%	11.1%	11.1%
$uP_{CTNNB1\_A*03/A*11\_1}$	EVYHTTVLK	A*03/A*11	CTNNB1 NM_001904 UUG.1	high frequent	10.0%	11.1%	8.9%
uP_{MAPK6_B*07}	SPTLNVSAL	B*07	MAPK6 NM_002748.4 AUG.2, AUG.3	high frequent	8.9%	11.1%	6.7%
$uP_{RUNX1\_A*03/A*11}$	SSALLQLLK	A*03/A*11	RUNX1 NM_001001890 AUG.6	high frequent	5.6%	6.7%	4.4%
$uP_{ASNSD1\_B*40\_1}$	SESKNILDEL	B*40	ASNSD1 NM_019048 AUG.3, UUG.2, AUG.5	reviewed	2.2%	2.2%	2.2%

HLA uLigands selected for validation with synthetic peptides. Abbreviations: ID, identification; freq., frequency.

Dataset S1 (separate file). Identified uORF-derived HLA ligands with sequence, HLA restriction, and source uORF.

References

1. Wethmar, K. *et al.* Comprehensive translational control of tyrosine kinase expression by upstream open reading frames. *Oncogene* **35**, 1736–1742 (2016).
2. McGillivray, P. *et al.* A comprehensive catalog of predicted functional upstream open reading frames in humans. *Nucleic Acids Research* **46**, 3326–3338 (2018).
3. Hernández, G., Osnaya, V. G. & Pérez-Martínez, X. Conservation and Variability of the AUG Initiation Codon Context in Eukaryotes. *Trends in Biochemical Sciences* **44**, 1009–1021 (2019).
